# Supplementary material for: Tuning evolvability via plasmid copy number and regulatory architecture
Source: Nat Commun. 2025 Dec 31;17:1230. doi: 10.1038/s41467-025-67995-9 (PMC12864740; doi:10.1038/s41467-025-67995-9)
Supplement: Supplementary file 1 — Supplementary Information [file 41467_2025_67995_MOESM1_ESM.pdf]

Supplementary Information

Ximing Li,[1] Andras Gyorgy[1*]

[1]Division of Engineering, New York University Abu Dhabi, Abu Dhabi, UAE
*To whom correspondence should be addressed; E-mail: andras.gyorgy@nyu.edu.

This Supplementary Information provides further details about (i) the distribution and emergence of mutations and plasmid heterogeneity, and how plasmid copy number (PCN) affect them; (ii) the EvolvR system, focusing on its metabolic burden and targeting accuracy considering both plasmid and genome sequencing data to quantify potential off-target effects; (iii) gating, mutant detection, and our estimation of the phenotypic mutation rate based on fluctuation analysis; (iv) the IFFL-based control module to ensure that the expression level of the reporter protein remains unaffected by variations in PCN; (v) LacI expression with and without self-repression and the corresponding metabolic burden; (vi) flow cytometer data and phenotypic mutation rate estimates together with statistical significance tests; and (vii) plasmid maps and sequences of the constructs featured in the manuscript.

Contents

1 Distribution and emergence of mutations and plasmid heterogeneity 2

2 Metabolic burden and targeting accuracy of EvolvR 4

3 Gating, mutant detection, and phenotypic mutation rate estimation 10

4 IFFL-based control module 12

5 Constitutive and self-repressed LacI expression 14

6 Flow cytometer data, phenotypic mutation rates and statistical significance tests 17

7 Plasmid maps and sequences 29

1 Distribution and emergence of mutations and plasmid heterogeneity

Data presented in Fig. 1 and Supplementary Fig. 1 reveal that the main observations are not sensitive to changes in the parameter values of the stochastic model (number of generations, population size, and mutation rate), namely: (i) the number of mutant bacteria increases with PCN; and (ii) the average fraction of mutated plasmids per mutant bacteria decreases with PCN.

To estimate the distribution of genetic variations at the plasmid level, we first note that EvolvR mutates the target at a rate of $2.5 \cdot 10^{-6}$ per nucleotide per generation [1], suggesting that mutations are sparse across generations. This aligns well with fluctuation analysis [2], where a few early-generation mutations expand into descendants. Consequently, many mutant

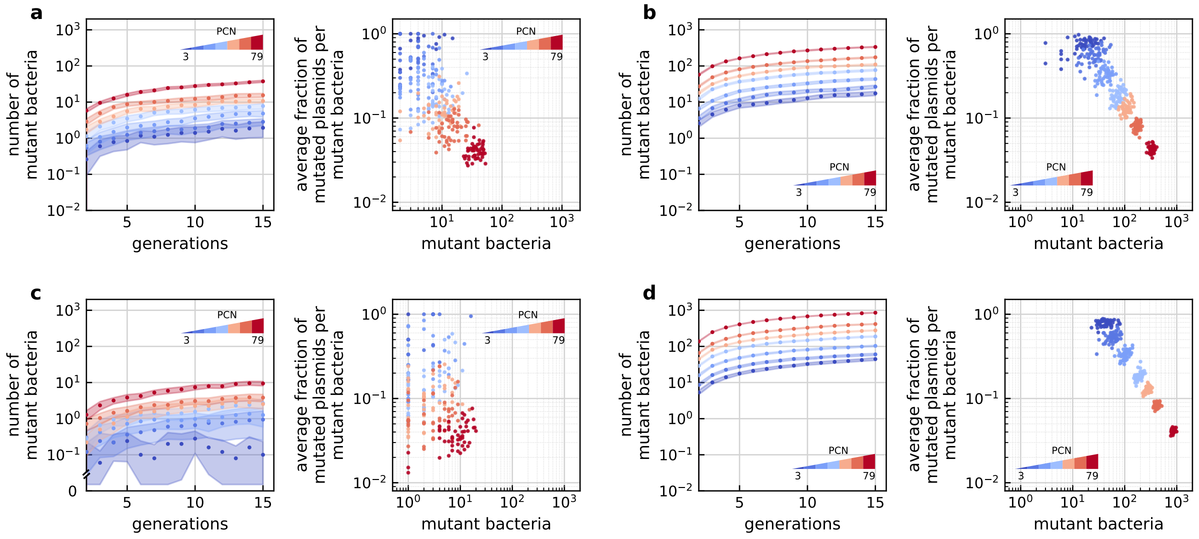

Supplementary Fig. 1. **The qualitative relationship between PCN and mutations is insensitive to changes in model parameters.** Unless otherwise noted, in all panels the mutation rate is $\mu = 2.5 \cdot 10^{-6}$ per nucleotide per generation, the population size is $N = 10^5$ and simulations are performed for 15 generations. Selected values of PCN correspond to the average for the pSC101 variants used in subsequent genetic constructs (i.e., 3, 5, 9, 17, 25, 39, and 79). For each PCN, points represent mean values from 50 independent replicates and shaded areas represent 95% confidence intervals. **a** The mutation rate is $\mu = 10^{-6}$. **b** The mutation rate is $\mu = 10^{-5}$. **c** The population size is $N = 10^4$. **d** The population size is $N = 10^6$.

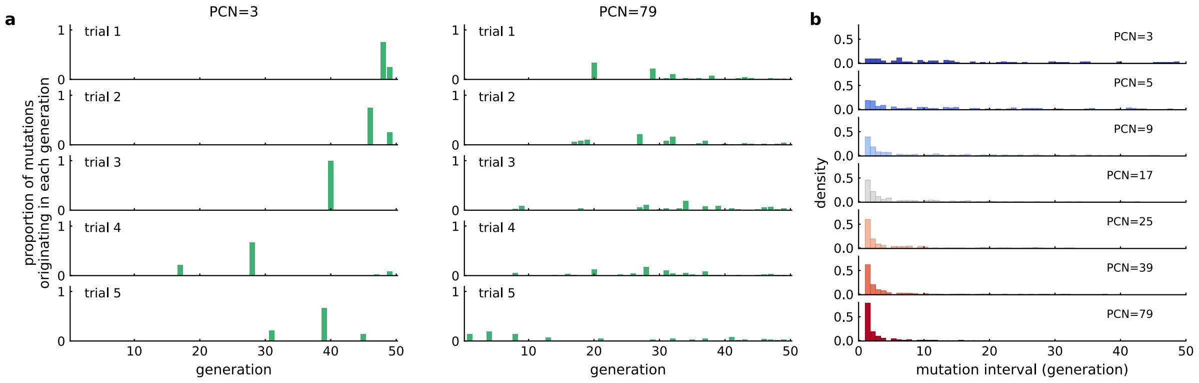

Supplementary Fig. 2. **Temporal distribution of the emergence of distinct mutations.** In the final (50th) generation, mutant plasmids are evaluated based on the generation in which their mutations first appeared. Each colored bar indicates the proportion of mutations originating from a specific generation among all mutant plasmids. For instance, a bar of height 0.7 at generation 15 signifies that 70% of the mutant plasmids in generation 50 have mutations that emerged in generation 15. **a** For low copy plasmids, with an average PCN of 3, mutations occurred sparsely (few tall bars in each simulation trial). Conversely, for high copy plasmids, with an average PCN of 79, mutations occurred frequently (multiple short bars in each simulation trial). **b** Mutation intervals are derived as the differences of generations between consecutive mutation events for each simulation trial. Distributions are obtained from 50 independent trials for each PCN. Short intervals indicate frequent mutation events, whereas long intervals suggest sparse mutations. Mutations are rare at low PCNs and become more frequent as PCN increases.

plasmids are expected to share similar genotypes from a few mutated ancestors that arise early on. To investigate the issue of intrinsic plasmid heterogeneity, we categorized mutated plasmids based on the generation in which each mutation first occurred. For simplicity, we assume that each plasmid mutates at most once, as the EvolvR-associated sgRNA loses binding affinity to a mutated target. Thus, each mutated plasmid is assigned to a unique generation. The distribution of these generations (that eventually gives rise to the distinct mutations we observe in the final simulated generation) is presented in Supplementary Fig. 2. As expected, mutations that arise in early generations tend to dominate the final mutant population, especially in low copy number plasmids (Supplementary Fig. 2). In contrast, high copy number plasmids exhibit greater genotypic diversity, with mutations emerging across multiple generations (Supplementary Fig. 2).

2 Metabolic burden and targeting accuracy of EvolvR

Here we review the performance of the EvolvR system. In particular, we first characterize its expression profile upon arabinose induction and the metabolic burden its host faces. Following this, to assess the impact and accuracy of EvolvR, we present both plasmid and genome sequencing data to quantify potential off-target effects, in addition to intrinsic genetic variation due to the basal mutation of bacterial cells [1].

2.1 Induction with arabinose and metabolic burden

We utilize a P_{BAD}-inducible system to tightly regulate EvolvR and sgRNA expression [3]. To determine the activity of the P_{BAD} promoter, we characterized a sensor circuit that produces Enhanced Yellow Fluorescent Protein (EYFP) upon arabinose induction, revealing that concentrations above 2 mM correspond to saturating levels (Supplementary Fig. 3). Data in Supplementary Fig. 4 highlight that (i) induction via arabinose in the absence of EvolvR on the p15A plasmid or harboring the target plasmid expressing $sfGFP_d$ from a pSC101 plasmid do not impact cell growth; whereas (ii) the expression of EvolvR from a p15A plasmid does.

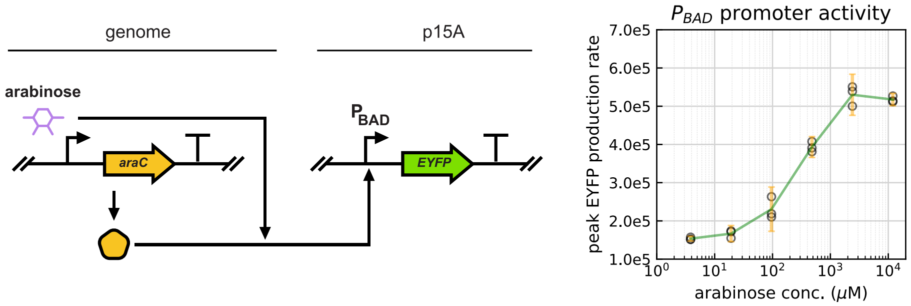

Supplementary Fig. 3. **The P_{BAD} promoter regulating EvolvR activity responds to arabinose induction.** AraC is constitutively expressed from the genome. *E. coli* DH5α cells harboring the circuit were grown at 30°C for 22 hours under conditions identical to EvolvR induction (see Methods). Data were obtained in microplate reader experiments considering 3 independent replicates. Points represent mean values and error bars indicate 95% confidence intervals.

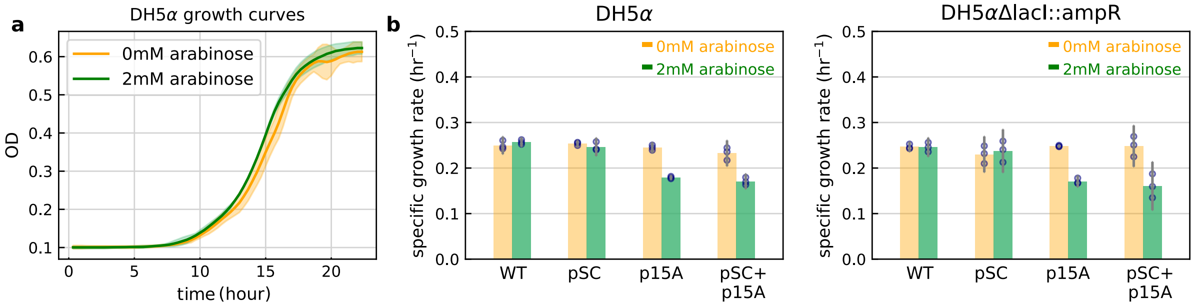

Supplementary Fig. 4. **Effects of arabinose induced EvolvR expression on host cell fitness.** In all experiments, cells were grown at 30°C for 22 hours under conditions identical to EvolvR induction (see Methods). Data were obtained in microplate reader experiments considering 3 independent replicates. Points represent means values. Shading or error bars indicate 95% confidence intervals. **a** Typical growth curves of DH5α cells carrying no plasmids with and without 2 mM arabinose induction. **b** DH5α or DH5$\alpha\Delta$lacI::ampR cells harbor one of the following: (i) no plasmid ("WT"); (ii) pSC101 plasmid constitutively expressing sfGFP$_d$ from Fig. 2a ("pSC"); (iii) p15A plasmid harboring EvolvR and sgRNA from Fig. 2a ("p15A"); or (iv) both plasmids ("pSC+p15A").

2.2 Plasmid-borne mutations

Since next-generation sequencing (NGS) measurements characterize mutation genotypes that emerge only in bulk at the population level, we isolated individual colonies exhibiting mutant phenotypes and prepared the plasmids for sequencing. In particular, cells were grown after arabinose induction, and following regrowth, the ones showing sfGFP expression were sorted and regrown on agar plates (see Methods). Thus, plasmids in the resulting individual colonies are expected to be genetically homogeneous, hence sequencing them provides fine-grained genetic information that is not available via bulk NGS.

EvolvR is designed to edit within a window that spans 60 nucleotides in the 3' direction from the Cas9 nick site (light violet in Supplementary Fig. 5), with mutations potentially occurring beyond this range with decreasing efficiency [1]. The size of this window depends on the processivity of the error-prone DNA polymerase in EvolvR (PolI3M), and can be further modulated via the insertion of a thioredoxin-binding domain (TBD) into the polymerase (PolI3M-TBD).

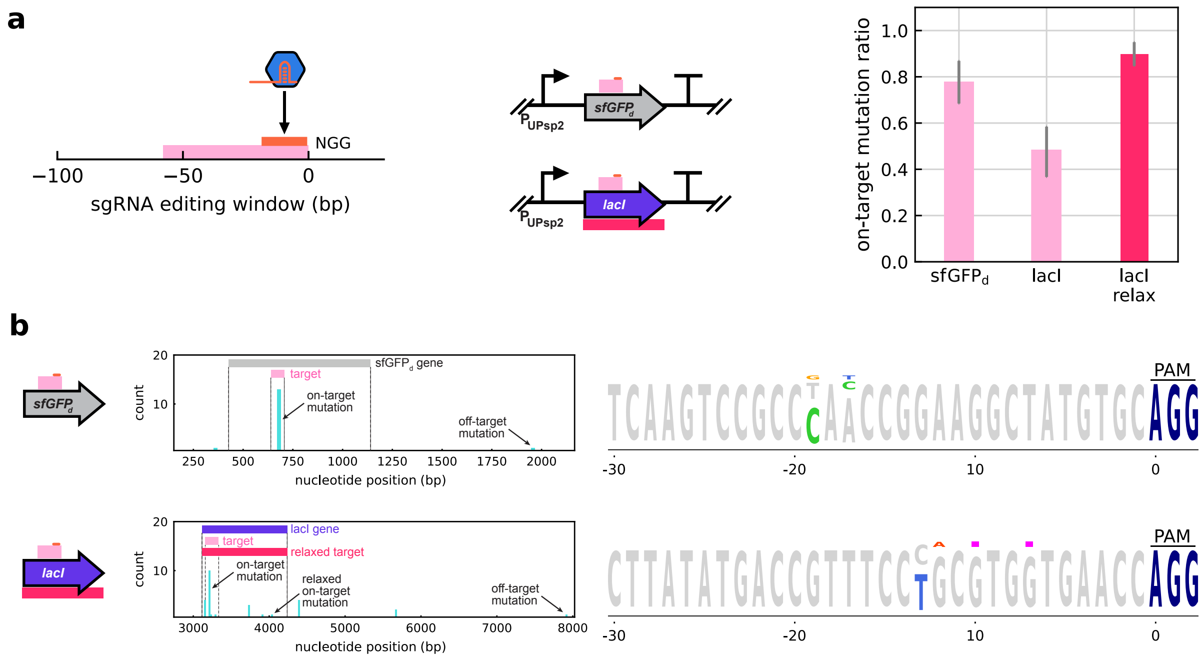

Supplementary Fig. 5. **Targeting efficiency of the EvolvR system.** For each sgRNA, individual colonies showing mutation phenotypes (i.e., high sfGFP expression) were collected for whole plasmid sequencing using the pSC101 plasmid with three copies per cell. Light violet denotes the intended editing window, dark violet corresponds to the relaxation covering the whole *lacI* gene. Cells are induced with 2 mM arabinose. **a** Efficiency of sgRNAs targeting $sfGFP_d$ and *lacI* (PCN=3). NGG indicates the PAM sequence. The sample size for $sfGFP_d$ and *lacI* targeting sgRNAs is 15 and 21, respectively. Bars represent mean on-target ratios and gray error bars indicate 95% confidence intervals. **b** Mutation counts are shown along the plasmid. Non-mutant nucleotides are depicted in gray within a 30-nucleotide editing window for the sgRNAs targeting $sfGFP_d$ and *lacI*, other colors indicate mutations. The size of each nucleotide character is proportional to its occurrence probability at the position. Sequences with on-target mutations are aligned for the sgRNA targeting $sfGFP_d$ and *lacI*.

Mutations that occur within the intended region are considered on-target, otherwise they are off-target. For each sequenced sample, on-target ratios are calculated by dividing the on-target mutation counts with respect to the total number of mutations. For the sgRNA targeting $sfGFP_d$, mutations occur predominantly within the target window, and off-target mutations become more prevalent in case of *lacI* (Supplementary Fig. 5a). Importantly, the majority of these unintended mutations are still restricted to the *lacI* gene, giving rise to the same mutation phenotype ($LacI_d$).

To capture this, a relaxed criterion was used to include these mutations by expanding the target window to encompass the entire *lacI* gene (dark violet in Supplementary Fig. 5).

Cas9-based systems are known to suffer from off-target effects, especially when binding

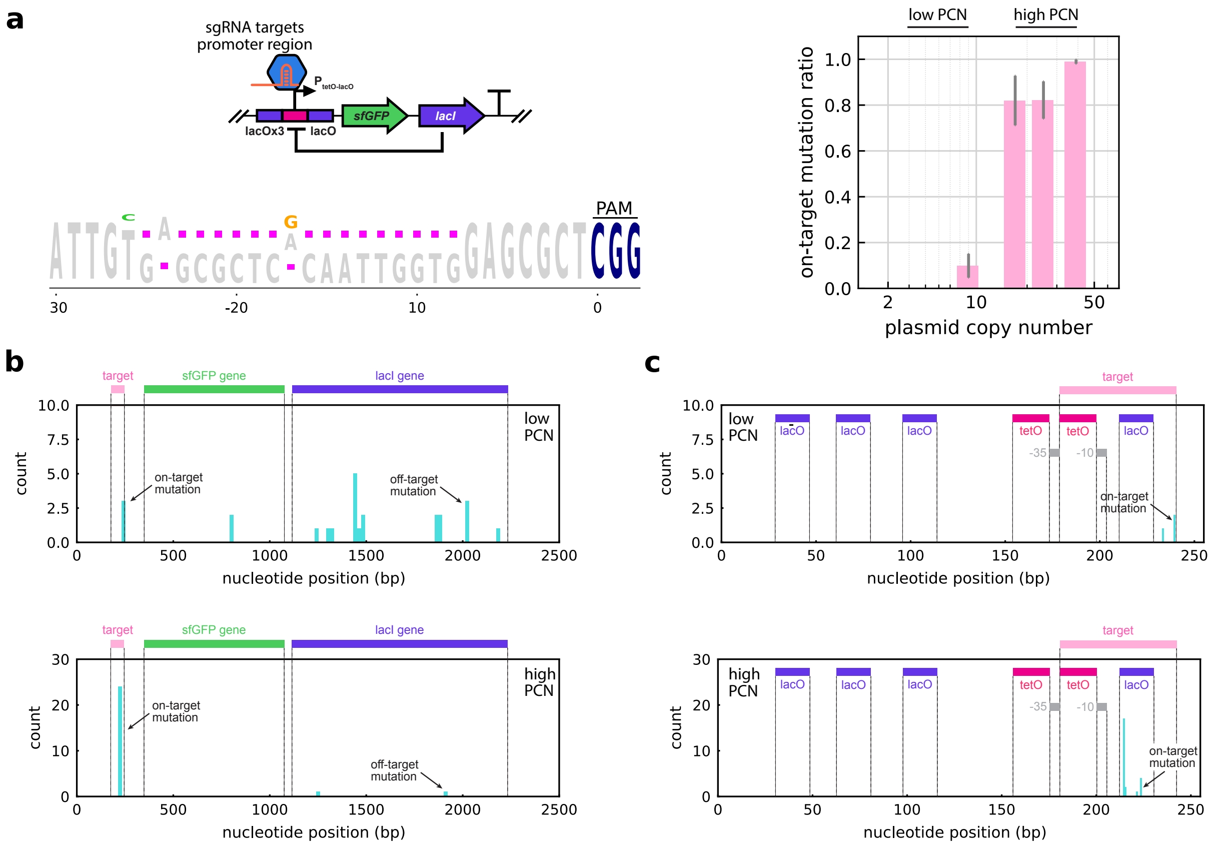

Supplementary Fig. 6. **Targeting efficiency of the EvolvR system depends on the relative abundance of EvolvR and targets.** For each sgRNA, individual colonies showing mutation phenotypes (i.e., high sfGFP expression) were collected for whole plasmid sequencing using pSC101 variants. Low PCN indicates data corresponding PCN values of 3 and 9, whereas high PCN represents data obtained with pSC101 variants with PCN of 17, 25, and 39. Cells are induced with 2 mM arabinose. **a** Efficiency of sgRNA targeting *lacO*. NGG indicates the PAM sequence. Sample size is 8 for all PCNs. Non-mutant nucleotides are depicted in gray within a 30-nucleotide editing window for the sgRNAs targeting *lacO*, other colors indicate mutations. The size of each nucleotide character is proportional to its occurrence probability at the position. Sequences with on-target mutations are aligned for the sgRNA targeting *lacO*. The exact alignment pattern depends on the aligning algorithm and parameters. Bars represent mean on-target ratios and gray error bars indicate 95% confidence intervals. **b** Mutation counts along the plasmid. **c** Mutation counts around the promoter region.

sites become saturated due to the high relative prevalence of Cas9 with respect to its target [4]. Therefore, it is not surprising that we observe decreased EvolvR specificity for the sgRNA recognizing the *lacO* binding sites, especially when the PCN harboring the target sequence is low (Supplementary Fig. 6a). To understand why this happens, unlike when targeting $sfGFP_d$ or *lacI*, note that LacI binding to *lacO* blocks EvolvR from accessing the promoter [5], diverting it to unintended sites and resulting in off-target mutations, for instance, in the *lacI* gene (Supplementary Fig. 6bc). Inducers such as IPTG can relieve LacI from binding to *lacO*, however, this leads to constitutive sfGFP expression, which would accumulate over generations and interfere with downstream mutant detection using flow cytometry. Thus, we instead integrated a TetR repression module into the two-plasmid system to repress both LacI and sfGFP expression during EvolvR activation (Fig. 6a). By blocking LacI production this way, the *lacO* binding sites become accessible for EvolvR to initiate on-target mutations. Additionally, we lowered the expression level of EvolvR by using 200 μM arabinose instead of 2 mM to reduce the chance of EvolvR binding to off-target sites. Just as before, on-target mutations are found at a few specific positions that are functionally important, in this case, to disrupt the *lacO* binding sites, resulting in the dominance of a small subset of genotypes (Supplementary Fig. 6a).

2.3 Genomic mutations

E. coli has a basal spontaneous mutation rate of approximately 10^{-10} mutations per nucleotide per generation [1, 6]. The Cas9 variant in EvolvR (enCas9-PolI3M-TBD) is engineered so that it has reduced non-specific DNA binding, resulting in on-target and global mutation rates of approximately 10^{-5} and 10^{-8} mutations per nucleotide per generation, respectively [1]. We utilized a P_{BAD}-inducible system to tightly regulate EvolvR and sgRNA expression [3], hence the approximate background mutation rate can be estimated as follows.

Prior to induction, cultures are grown for around 24 hours (i.e., 32 generations at 37°C) on

Supplementary Table 1. **Genomic variant detection.** Variants with quality score over 20 and read depth (RD) over 10 are considered valid [8]. DH5$\alpha\Delta$lacI::ampR is used for constructs with *lacI* to avoid the unwanted targeting of genomic *lacI*. The only detected mutation occurs when the sgRNA targets *sfGFP$_d$*, yielding a single nucleotide polymorphism (A to T) in the *PqqL* gene encoding for a zinc protease (turning the 122nd amino acid from Val to Glu; no nearby sequence resembles the sgRNA target, but several NGG motifs are present). High copy plasmid is used for *ampR$_d$* as the number of mutant bacteria increases with PCN (Fig. 3d).

sgRNA target	Strain	PCN	Highest Quality	RD	Variant Detected
–	DH5α	–	2.12e-10	8	No
–	DH5$\alpha\Delta$lacI::ampR	–	4.0e-14	150	No
sfGFP$_d$	DH5α	3	5522.84	168	Yes
lacI	DH5$\alpha\Delta$lacI::ampR	3	4.89e-14	272	No
lacO	DH5$\alpha\Delta$lacI::ampR	3	8.65e-14	150	No
ampR$_d$	DH5α	79	6.78	4	No

agar (12 hours) and liquid media (12 hours) with 0.2% glucose (Fig. 2b) to suppress basal P_{BAD} expression through catabolite repression, ensuring a low spontaneous mutation rate during this stage. Subsequently, we induce EvolvR expression using arabinose for 22 hrs at 30°C, i.e., for approximately 15 generations. Considering the typical *E. coli* genome size, this is expected to generate approximately $5 \cdot 10^6 \cdot (10^{-10} \cdot 32 + 10^{-8} \cdot 15) = 0.766$ mutations, i.e., less than a single mutation throughout the experiment (and even fewer for the plasmids considering their maximal size and PCN: $13 \cdot 12 \cdot 10^3 \cdot (10^{-10} \cdot 32 + 10^{-8} \cdot 15) = 0.024$ for p15A with 13 copies [7] and $100 \cdot 8 \cdot 10^3 \cdot (10^{-10} \cdot 32 + 10^{-8} \cdot 15) = 0.123$ for pSC101 with no more than 100 copies).

To validate this estimate, we collected mutated samples after arabinose induction and performed genome sequencing to identify potential mutations (targeting *sfGFP$_d$*, *ampR$_d$*, *lacI*, and *lacO*). Following this, we performed variant calling on the genome sequences obtained using next generation sequencing (see Methods). Among all tested samples, only a single mutation was detected in the genome, for the sgRNA targeting *sfGFP$_d$* (Supplementary Table 1). These data confirm the low background mutation level suggested by our computational estimate.

3 Gating, mutant detection, and phenotypic mutation rate estimation

Flow cytometer data were gated using a density-based method [9] in two channels (Supplementary Fig. 7a). Subsequently, doublets were removed by excluding events with extreme area-to-height ratios (Supplementary Fig. 7b).

We use the sfGFP height signal (GFP-H) as a proxy for sfGFP expression (as it appears to be more consistent across flow conditions and less sensitive to variations in flow speed and cell size than the area signal GFP-A). To detect mutants with high sfGFP expression, we use the GFP-H distribution from the pre-induced culture as a reference. Fluorescence measurement via flow cytometer is often analyzed in the logarithmic scale [10, 11]. Accordingly, we assume that the data resemble a normal distribution in the logarithmic domain and apply a log-transformation prior to analysis. A threshold is defined on the log-transformed scale corresponding to a significance level of 0.01 using a standard one-sided t-test (Supplementary Fig. 8a). Events in the post-induced culture with GFP-H values over the threshold are identified as mutants (green dots in Supplementary Fig. 8b).

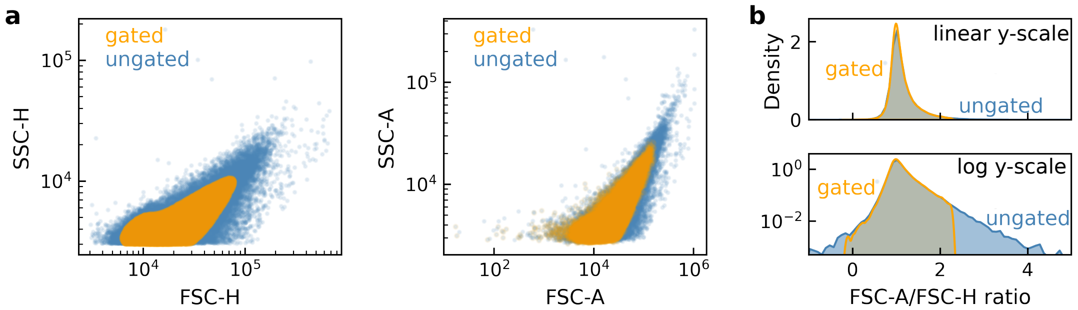

Supplementary Fig. 7. **Density-based gating.** The gated population represents 90% of the total events. **a** Flow cytometer data are gated based on density of events in forward scatter height (FSC-H) and side scatter height (SSC-H). **b** Doublets are filtered out as events with area-to-height ratios (FSC-A/FSC-H) more extreme than three standard deviations from the mean. These events are rare and become visible only when the density distribution of area-to-height ratios is displayed on a logarithmic scale [10, 11].

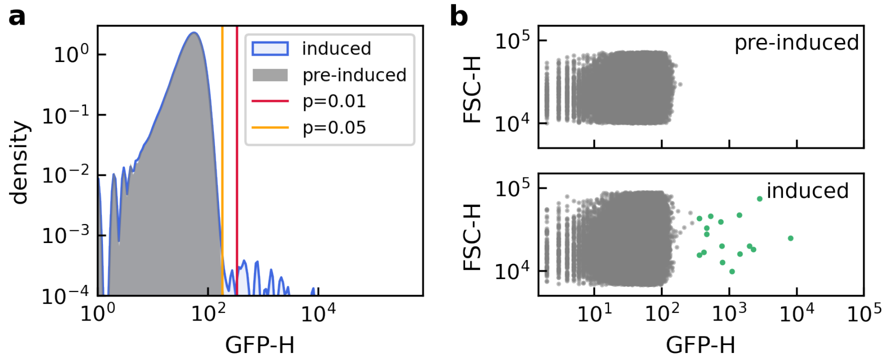

Supplementary Fig. 8. **Critical value-based identification of mutant subpopulation.** Cultures were screened prior to arabinose induction as a control to exclude spontaneously mutated individuals and to provide a reference for mutant detection. **a** Mutant events are identified based on the GFP-H distributions on a logarithmic scale using cutoff levels (yellow and red lines) derived from one-sided t-tests. **b** Events that exceed the cutoff levels are considered mutants (green dots).

Fluctuation analysis [2] is widely used to estimate mutation rates from replicate cultures that evolve in parallel. It assumes that mutations arise sporadically and independently in each culture following a Poisson distribution in each generation. The number of accumulated mutants by the final generation can be described by a Luria-Delbrück distribution. This compound Possion distribution typically exhibits high variability, from which the per-generation mutation rate can be estimated, for instance, using the probability generating function (PGF) model [12].

In our study, each PCN condition includes 8 independent replicate cultures, which were measured via flow cytometer to count mutants after induction. The distribution of mutant counts was subjected to fluctuation analysis to estimate the phenotypic mutation rate using the PGF model. The analysis was performed using the R package flan [12], specifically, the *mutestim* function assuming constant bacteria lifetimes. The estimated mean and 95% confidence intervals of the mutation rates are reported in the main figures and in Supplementary Figs. 11–18, where we also include the estimated statistical significance of the different phenotypic mutation rates across PCN conditions based on pairwise one-sided t-tests using the function *flan.test*.

4 IFFL-based control module

Considering first the circuit in Fig. 3a, let z_d and z denote the concentration of $\mathrm{sfGFP_d}$ and sfGFP, respectively. Let α_z further denote the total production rate constant of z and z_d combined (from the $\mathrm{P_{UPsp2}}$ promoter), whereas λ is their decay rate (combining degradation and dilution due to growth). With this, their dynamics are given by

$$\dot{z}_d = \alpha_z \frac{P - x}{P} - \lambda z_d, \qquad \dot{z} = \alpha_z \frac{x}{P} - \lambda z,$$

where P is the PCN and x denotes the number of mutated plasmids. Introducing

$$z_T = z_d + z,$$

we obtain

$$\dot{z}_T = \alpha_z - \lambda z_T,$$

that is, the total production rate of sfGFP and $\mathrm{sfGFP_d}$ is independent of the PCN due to the IFFL-based control module [13]. This is experimentally verified in Supplementary Fig. 9.

With

$$r = \frac{x}{P}$$

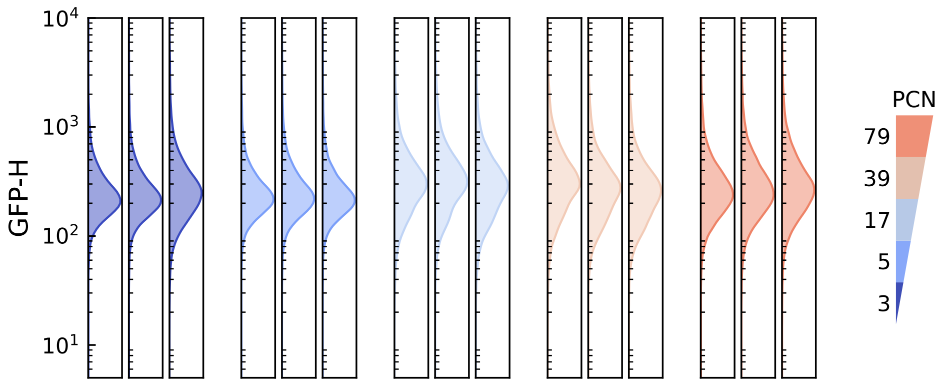

Supplementary Fig. 9. **The IFFL-based control circuit ensures sfGFP expression is independent of PCN.** Data obtained in flow cytometer experiments (in the absence of the p15A plasmid).

to capture the fraction of mutated plasmids, at the steady state we obtain

$$z = \frac{\alpha_z r}{\lambda}.$$

Thus, the concentration of functional sfGFP increases linearly with r (Fig. 3b). Considering parameter values typical in *E. coli* [14], we choose $\alpha_z = 125$ nM h^{-1} and $\lambda = 1$ h^{-1} in Fig. 3bc.

For the circuit in Fig. 4a, let z and z_d denote the concentration of LacI and LacI$_\mathrm{d}$, respectively. Using the same notation as before, and considering that sfGFP expression on the p15A plasmid is repressed by LacI, we have

$$\dot{z} = \alpha_z \frac{P - x}{P} - \lambda z_d, \qquad \dot{z}_d = \alpha_z \frac{x}{P} - \lambda z, \qquad \dot{y} = \alpha_y \frac{1}{1 + \frac{z}{K} + \frac{z_d}{K_d}} - \lambda y,$$

where α_y is the maximal production rate of sfGFP expression, whereas K and K_d denote the dissociation constant of LacI and LacI$_\mathrm{d}$ binding to their cognate promoter, respectively. Therefore, at the steady state we obtain

$$z = \frac{\alpha_z}{\lambda}\left(1 - r\right), \qquad z_d = \frac{\alpha_z}{\lambda} r, \qquad y = \frac{\alpha_y}{\lambda} \frac{1}{1 + \frac{\alpha_z}{\lambda}\frac{1-r}{K} + \frac{\alpha_z}{\lambda}\frac{r}{K_d}}.$$

The binding affinity of a mutated repressor to its cognate promoter is considerably lower than that of the wild-type variant (i.e., $K_d \gg K$). In the special case when binding is completely eliminated as a result of a mutation, we have $K_d \to \infty$, yielding

$$y = \frac{\alpha_y}{\lambda} \frac{1}{1 + \frac{\alpha_z}{\lambda}\frac{1-r}{K}},$$

to reveal that sfGFP expression decreases as the fraction r of mutated plasmid increases. Considering parameter values typical in *E. coli* [14], we choose $\alpha_y = 200$ nM h^{-1}, $\alpha_z = 125$ nM h^{-1}, $\lambda = 1$ h^{-1}, and $K = 1$ nM in Fig. 4c.

5 Constitutive and self-repressed LacI expression

After presenting the mathematical model for the circuits in Fig. 5, here we explore the metabolic burden of LacI production when it is constitutively expressed and when it is self-repressed.

5.1 Constitutive LacI expression

Considering first the circuit in Fig. 5a when targeting *lacI*, let z, z_d, and y denote the concentration of LacI, $\mathrm{LacI_d}$ (mutated variant), and sfGFP, respectively. Furthermore, let β_z denote the production rate constant of LacI/$\mathrm{LacI_d}$ from a single plasmid using the $\mathrm{P_{const}}$ promoter, whereas β_y represents the maximal production rate constant of sfGFP from a single plasmid using the $\mathrm{P_{lacO}}$ promoter, respectively (previously, α_z and α_y represented the total production rate constant from all promoters within a cell). As before, K and K_d denote the dissociation constant of LacI and $\mathrm{LacI_d}$ binding to the $\mathrm{P_{lacO}}$ promoter, respectively. With this, the dynamics of the system in Fig. 5a are given by

$$\dot{z} = \beta_z P\left(1-r\right) - \lambda z, \qquad \dot{z}_d = \beta_z P r - \lambda z_d, \qquad \dot{y} = \beta_y P \frac{1}{1+\frac{z}{K}+\frac{z_d}{K_d}} - \lambda y.$$

Therefore, at the steady state we obtain

$$z = \frac{\beta_z}{\lambda} P\left(1-r\right), \qquad z_d = \frac{\beta_z}{\lambda} P r, \qquad y = \frac{\beta_y P}{\lambda} \frac{1}{1+\frac{\beta_z P}{\lambda}\frac{1-r}{K}+\frac{\beta_z P}{\lambda}\frac{r}{K_d}},$$

so that when $K_d \to \infty$ (i.e., $\mathrm{LacI_d}$ loses its affinity to the $\mathrm{P_{lacO}}$ promoter), we have

$$y = \frac{\beta_y P}{\lambda} \frac{1}{1+\frac{\beta_z P}{\lambda}\frac{1-r}{K}}.$$

Considering parameter values typical in *E. coli* [14], we choose $\beta_z = 20$ nM h^{-1}, $\lambda = 1$ h^{-1} and $K = 1$ nM in Fig. 5a. To estimate β_y, we assume that $\mathrm{P_{lacO}}$ and $\mathrm{P_{const}}$ have comparable activity. We evaluate the translation rate of LacI relative to sfGFP using the RBS calculator [15], estimating that the latter has a translation rate approximately 9 times of the former. Therefore we choose $\beta_y = 2.5$ nM h^{-1} in Fig. 5a.

For the circuit in Fig. 5a when targeting the P_{lacO} promoter, let z and y denote the concentration of LacI and sfGFP, respectively. Mutations in the P_{lacO} promoter result in P_{lacOd}, increasing the dissociation constant of LacI to the promoter from K to K_d [16]. As a result, the dynamics of the system are given by

$$\dot{z} = \beta_z P - \lambda z, \qquad \dot{y} = \beta_y P \left(\frac{1-r}{1+\frac{z}{K}} + \frac{r}{1+\frac{z}{K_d}} \right) - \lambda y,$$

so that at the steady sate we have

$$z = \frac{\beta_z}{\lambda} P, \qquad y = \frac{\beta_y P}{\lambda} \left(\frac{1-r}{1+\frac{\beta_z P}{\lambda K}} + \frac{r}{1+\frac{\beta_z P}{\lambda K_d}} \right) \approx \frac{\beta_y P}{\lambda} \left(\frac{1-r}{1+\frac{\beta_z P}{\lambda K}} + r \right)$$

when $K_d \to \infty$ (i.e., LacI loses its affinity to the P_{lacOd} promoter). As in the previous case, we choose $\beta_z = 20$ nM h^{-1}, $\beta_y = 2.5$ nM h^{-1}, $\lambda = 1$ h^{-1}, and $K = 1$ nM.

5.2 Self-repressed LacI expression

For the circuit in Fig. 5b when targeting *lacI*, let z, z_d, and y denote the concentration of LacI, LacI_{d} (mutated variant), and sfGFP, respectively. Furthermore, let β_z and β_y denote the maximal production rate constant of LacI/LacI_{d} and sfGFP from a single plasmid. Therefore, the dynamics of the system are given by

$$\dot{z} = \beta_z P \frac{1-r}{1+\frac{z}{K}+\frac{z_d}{K_d}} - \lambda z, \qquad \dot{z}_d = \beta_z P \frac{r}{1+\frac{z}{K}+\frac{z_d}{K_d}} - \lambda z_d, \qquad \dot{y} = \beta_y P \frac{1}{1+\frac{z}{K}+\frac{z_d}{K_d}} - \lambda y.$$

Assuming that $K_d \to \infty$ (i.e., LacI_{d} loses its affinity to the P_{lacO} promoter), at the steady state we have

$$z = \frac{-K + \sqrt{K^2 + 4(1-r)\frac{\beta_z P K}{\lambda}}}{2}, \qquad z_d = \frac{\beta_z P}{\lambda} \frac{r}{1+\frac{z}{K}}, \qquad y = \frac{\beta_y P}{\lambda} \frac{1}{1+\frac{z}{K}}.$$

For the circuit in Fig. 5b when targeting the P_{lacO} promoter, let z and y denote the concentration of LacI and sfGFP, respectively. Mutations in the P_{lacO} promoter result in P_{lacOd}, increasing

the dissociation constant of LacI to the promoter from K to K_d [16], yielding

$$\dot{z} = \beta_z P \left(\frac{1-r}{1+\frac{z}{K}} + \frac{r}{1+\frac{z}{K_d}} \right) - \lambda z, \qquad \dot{y} = \beta_y P \left(\frac{1-r}{1+\frac{z}{K}} + \frac{r}{1+\frac{z}{K_d}} \right) - \lambda y.$$

With $K_d \to \infty$ (i.e., LacI loses its affinity to the $\mathrm{P_{lacOd}}$ promoter), at the steady state we have

$$z = \frac{\left(\frac{\beta_z P r}{\lambda} - K\right) + \sqrt{\left(\frac{\beta_z P r}{\lambda} - K\right)^2 + 4\frac{\beta_z P K}{\lambda}}}{2}, \qquad y = \frac{\beta_y P}{\lambda} \left(\frac{1-r}{1+\frac{z}{K}} + r \right).$$

As before, we choose $\beta_z = 20$ nM h^{-1}, $\beta_y = 2.5$ nM h^{-1}, $\lambda = 1$ h^{-1}, and $K = 1$ nM.

5.3 Metabolic burden and genetic stability

Considering the impact of these constructs on the host, we observed that constitutive expression of LacI limits the PCN that can be reliably harbored by the host DH5$\alpha\Delta$lacI::ampR. Once the PCN reaches 25, the plasmid is prone to form dimers *in vivo*, and over this threshold, the construct becomes genetically unstable. This suggests that constitutively expressed LacI imposes considerable metabolic burden on the host (Supplementary Fig. 10), compromising its fitness, and that this effect is exacerbated as PCN increases. The same negative circuit-host interaction is absent for the construct with self-repressed LacI, as expected based on simulation data (Supplementary Fig. 10). As a result, while the former motif limits the PCN range, the latter does not impose such restrictions, thus providing a potential evolutionary advantage among other factors [17].

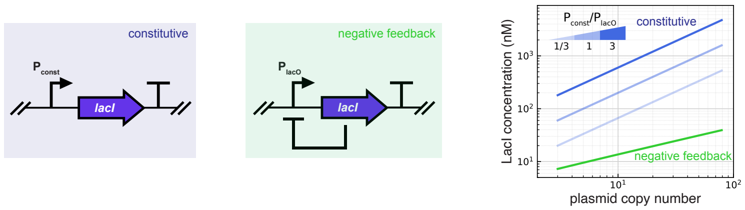

Supplementary Fig. 10. **Negative autoregulation of LacI reduces its expression level, thus the metabolic burden that it represents to the host.** Simulation parameters are identical to those used in Fig. 5. We choose $\beta_y = 20$ nM h^{-1} for $\mathrm{P_{lacO}}$, $\lambda = 1$ h^{-1}, and $K = 1$ nM for LacI binding to $\mathrm{P_{lacO}}$.

6 Flow cytometer data, phenotypic mutation rates and statistical significance tests

In Supplementary Figs. 11–18 we present data from 8 independent replicates for each PCN that underpin the experimental results featured in Figs. 2–6, alongside with the corresponding statistical significance tests using pairwise one-sided t-tests. The underlying flow cytometer scatterplots for both pre-induced and post-induced cultures are included in Supplementary Figs. 19–25. For Supplementary Fig. 13, fluctuation analysis is based on the number of mutant colonies that grow on agar plates with and without carbenicillin, such that for each pSC101 variant circuit data from approximately 50 independent replicates in total are collected, performed in 3 different trials.

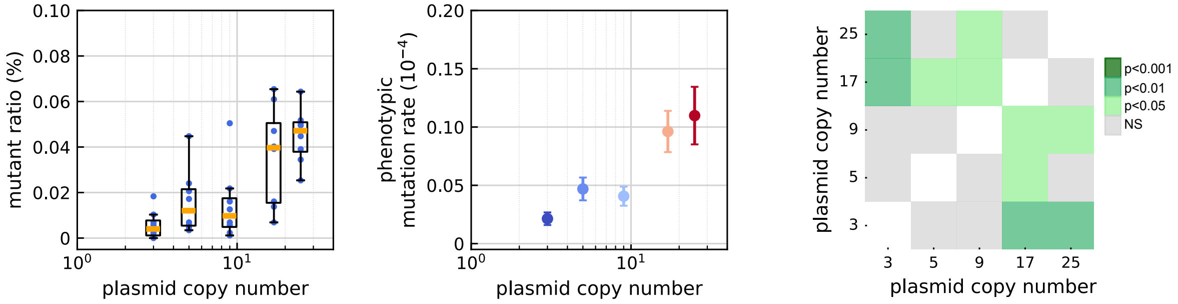

Supplementary Fig. 11. **Mutant ratio, phenotypic mutation rate and statistical significance test for the circuit in Fig. 2a.** For each PCN, experimentally measured mutant ratios from 8 independent replicates are used to estimate phenotypic mutation rates following fluctuation analysis. Centers represent median values in the boxplot figure and mean values in the phenotypic mutation rate figure. Error bars in the phenotypic mutation rate figure represent 95% confidence intervals. In the boxplot, the lower and upper bounds of the box represent the first quartile (Q1) and third quartile (Q3), respectively. With IQR = Q3-Q1 denoting the interquartile range, the whiskers extend from the box to the minimum and maximum non-outlier values such that the lower and upper thresholds are defined as Q1-1.5IQR and Q3+1.5IQR, respectively.

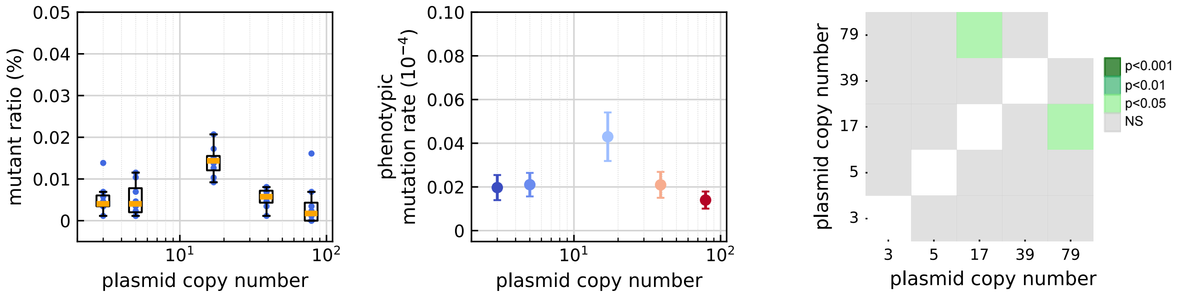

Supplementary Fig. 12. **Mutant ratio, phenotypic mutation rate and statistical significance test for the circuit in Fig. 3a.** For each PCN, experimentally measured mutant ratios from 8 independent replicates are used to estimate phenotypic mutation rates following fluctuation analysis. Centers represent median values in the boxplot figure and mean values in the phenotypic mutation rate figure. Error bars in the phenotypic mutation rate figure represent 95% confidence intervals. In the boxplot, the lower and upper bounds of the box represent the first quartile (Q1) and third quartile (Q3), respectively. With IQR = Q3-Q1 denoting the interquartile range, the whiskers extend from the box to the minimum and maximum non-outlier values such that the lower and upper thresholds are defined as Q1-1.5IQR and Q3+1.5IQR, respectively.

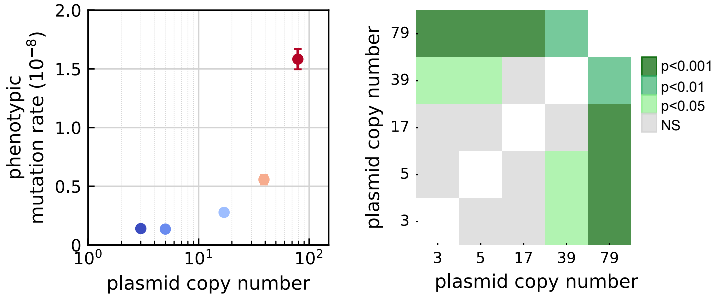

Supplementary Fig. 13. **Phenotypic mutation rate and statistical significance test for the circuit in Fig. 3e.** For each PCN, mutant colonies counted from 3 independent trials are used to estimate phenotypic mutation rates following fluctuation analysis. Error bars in the phenotypic mutation rate figure represent 95% confidence intervals.

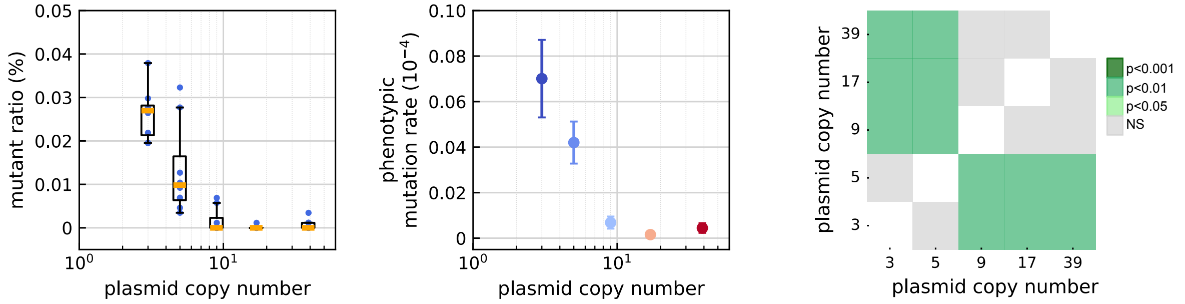

Supplementary Fig. 14. **Mutant ratio, phenotypic mutation rate and statistical significance test for the circuit in Fig. 4a.** For each PCN, experimentally measured mutant ratios from 8 independent replicates are used to estimate phenotypic mutation rates following fluctuation analysis. Centers represent median values in the boxplot figure and mean values in the phenotypic mutation rate figure. Error bars in the phenotypic mutation rate figure represent 95% confidence intervals. In the boxplot, the lower and upper bounds of the box represent the first quartile (Q1) and third quartile (Q3), respectively. With IQR = Q3-Q1 denoting the interquartile range, the whiskers extend from the box to the minimum and maximum non-outlier values such that the lower and upper thresholds are defined as Q1-1.5IQR and Q3+1.5IQR, respectively.

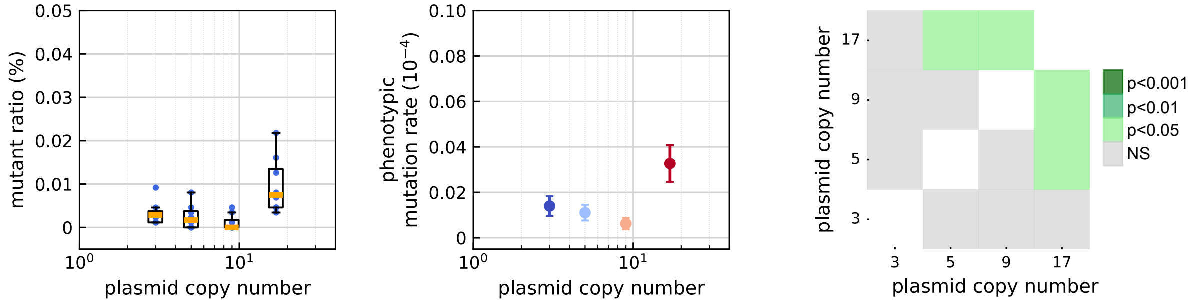

Supplementary Fig. 15. **Mutant ratio, phenotypic mutation rate and statistical significance test for the results in Fig. 6b (sgRNA targets the promoter region).** For each PCN, experimentally measured mutant ratios from 8 independent replicates are used to estimate phenotypic mutation rates following fluctuation analysis. Centers represent median values in the boxplot figure and mean values in the phenotypic mutation rate figure. Error bars in the phenotypic mutation rate figure represent 95% confidence intervals. In the boxplot, the lower and upper bounds of the box represent the first quartile (Q1) and third quartile (Q3), respectively. With IQR = Q3-Q1 denoting the interquartile range, the whiskers extend from the box to the minimum and maximum non-outlier values such that the lower and upper thresholds are defined as Q1-1.5IQR and Q3+1.5IQR, respectively.

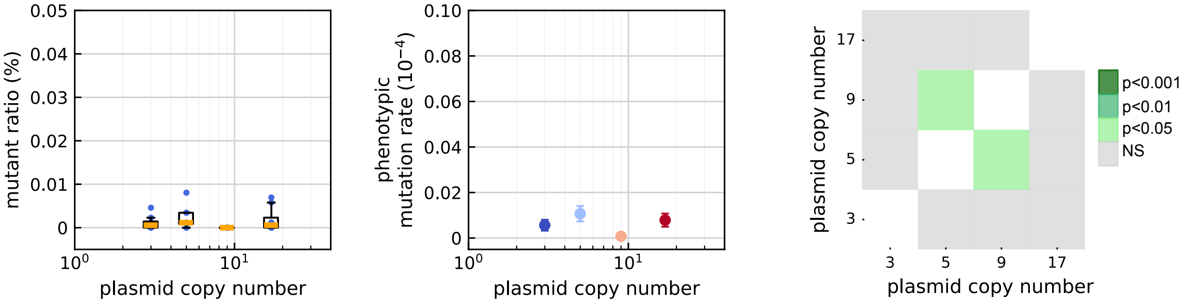

Supplementary Fig. 16. **Mutant ratio, phenotypic mutation rate and statistical significance test for the results in Fig. 6b (sgRNA targets the coding region).** For each PCN, experimentally measured mutant ratios from 8 independent replicates are used to estimate phenotypic mutation rates following fluctuation analysis. Centers represent median values in the boxplot figure and mean values in the phenotypic mutation rate figure. Error bars in the phenotypic mutation rate figure represent 95% confidence intervals. In the boxplot, the lower and upper bounds of the box represent the first quartile (Q1) and third quartile (Q3), respectively. With IQR = Q3-Q1 denoting the interquartile range, the whiskers extend from the box to the minimum and maximum non-outlier values such that the lower and upper thresholds are defined as Q1-1.5IQR and Q3+1.5IQR, respectively.

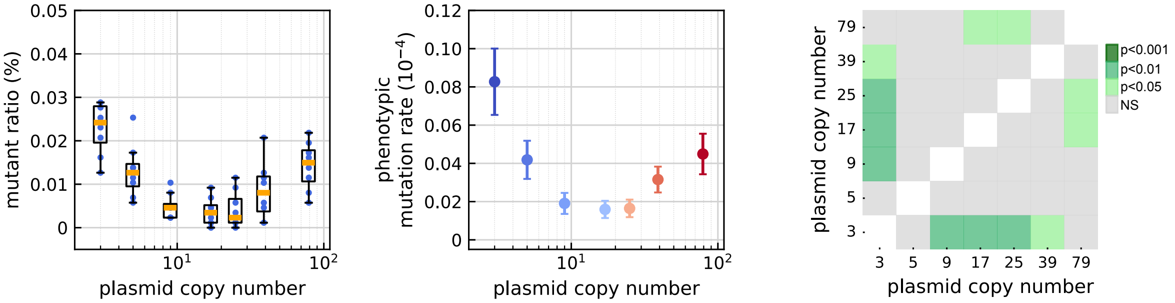

Supplementary Fig. 17. **Mutant ratio, phenotypic mutation rate and statistical significance test for the results in Fig. 6c (sgRNA targets the promoter region).** For each PCN, experimentally measured mutant ratios from 8 independent replicates are used to estimate phenotypic mutation rates following fluctuation analysis. Centers represent median values in the boxplot figure and mean values in the phenotypic mutation rate figure. Error bars in the phenotypic mutation rate figure represent 95% confidence intervals. In the boxplot, the lower and upper bounds of the box represent the first quartile (Q1) and third quartile (Q3), respectively. With IQR = Q3-Q1 denoting the interquartile range, the whiskers extend from the box to the minimum and maximum non-outlier values such that the lower and upper thresholds are defined as Q1-1.5IQR and Q3+1.5IQR, respectively.

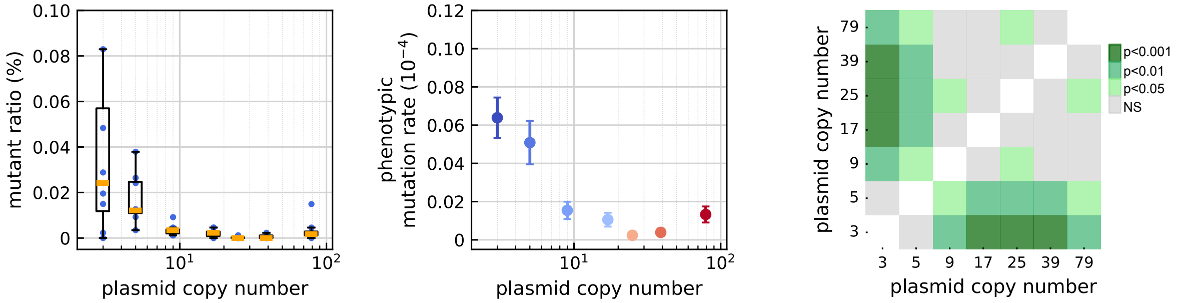

Supplementary Fig. 18. **Mutant ratio, phenotypic mutation rate and statistical significance test for the results in Fig. 6c (sgRNA targets the coding region).** For each PCN, experimentally measured mutant ratios from 8 independent replicates are used to estimate phenotypic mutation rates following fluctuation analysis. Centers represent median values in the boxplot figure and mean values in the phenotypic mutation rate figure. Error bars in the phenotypic mutation rate figure represent 95% confidence intervals. In the boxplot, the lower and upper bounds of the box represent the first quartile (Q1) and third quartile (Q3), respectively. With IQR = Q3-Q1 denoting the interquartile range, the whiskers extend from the box to the minimum and maximum non-outlier values such that the lower and upper thresholds are defined as Q1-1.5IQR and Q3+1.5IQR, respectively.

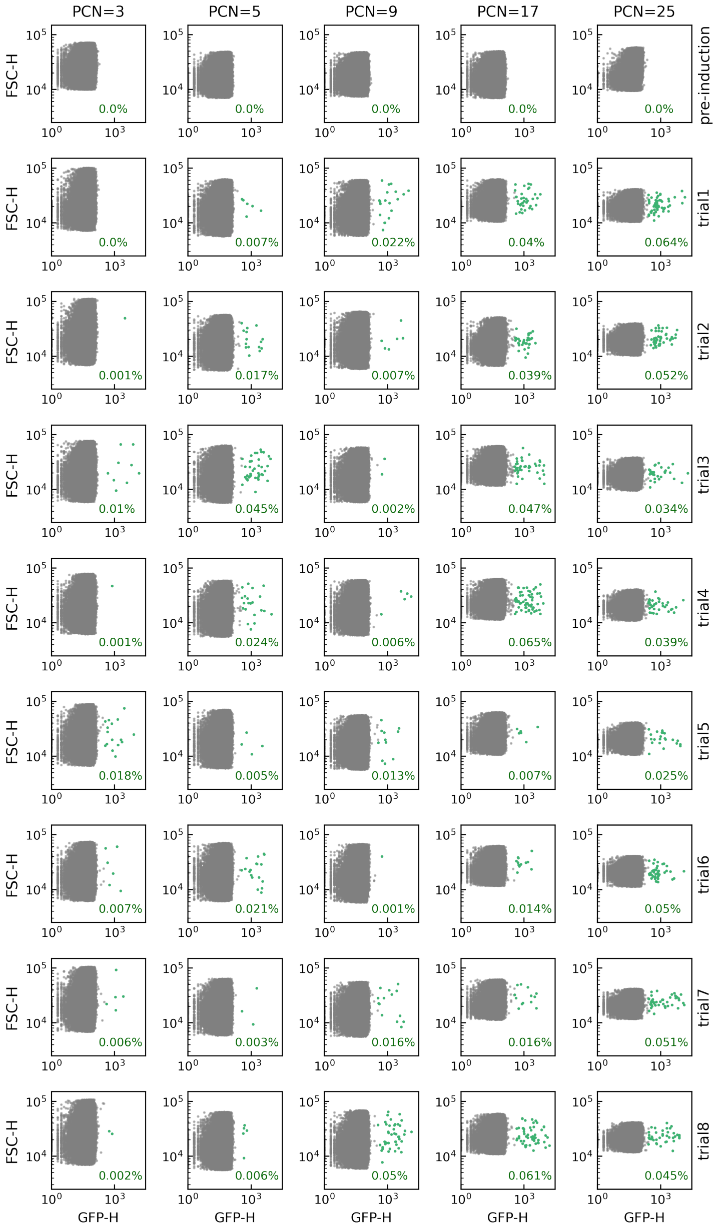

Supplementary Fig. 19. **Flow cytometer data for the circuit in Fig. 2a.** Green dots denote identified mutant events. Rows represent pre-induced culture and independent trials of post-induced cultures. Mutant ratios, labeled in green shown for each trial, correspond to the values in Supplementary Fig. 11. Pre-induced cultures with mutant ratios exceeding 0.01% were excluded from downstream procedures.

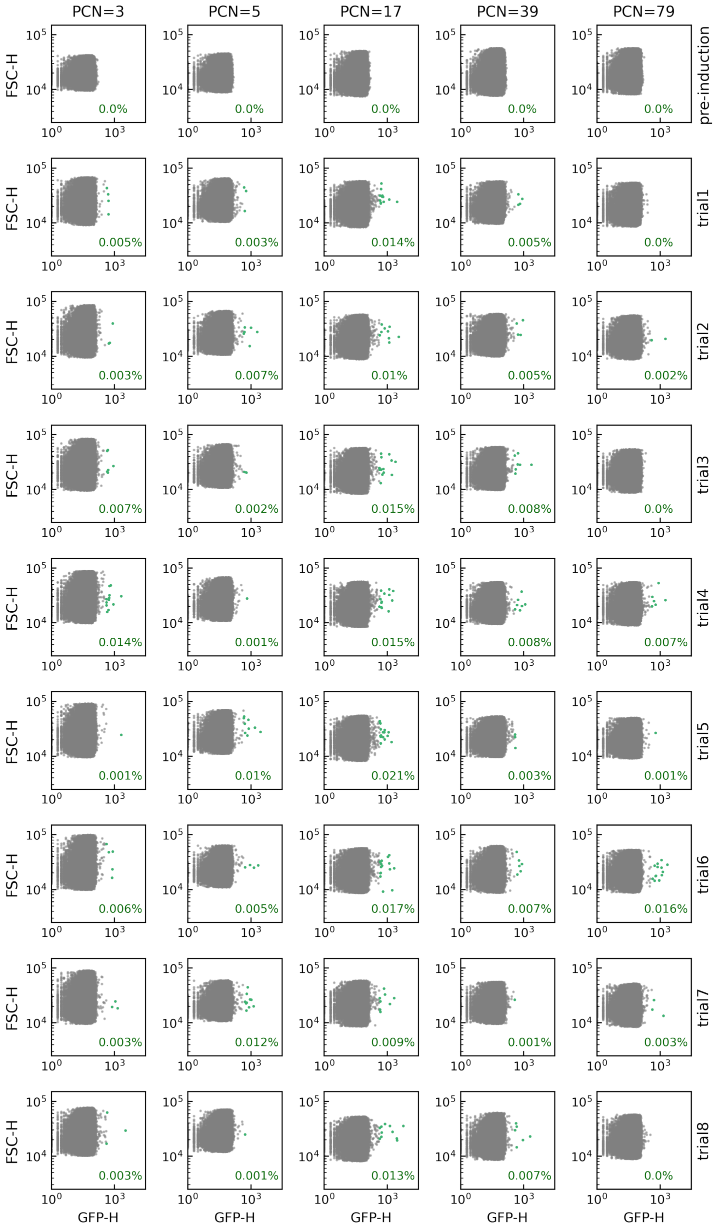

Supplementary Fig. 20. **Flow cytometer data for the circuit in Fig. 3a.** Green dots denote identified mutant events. Rows represent pre-induced culture and independent trials of post-induced cultures. Mutant ratios, labeled in green shown for each trial, correspond to the values in Supplementary Fig. 12. Pre-induced cultures with mutant ratios exceeding 0.01% were excluded from downstream procedures.

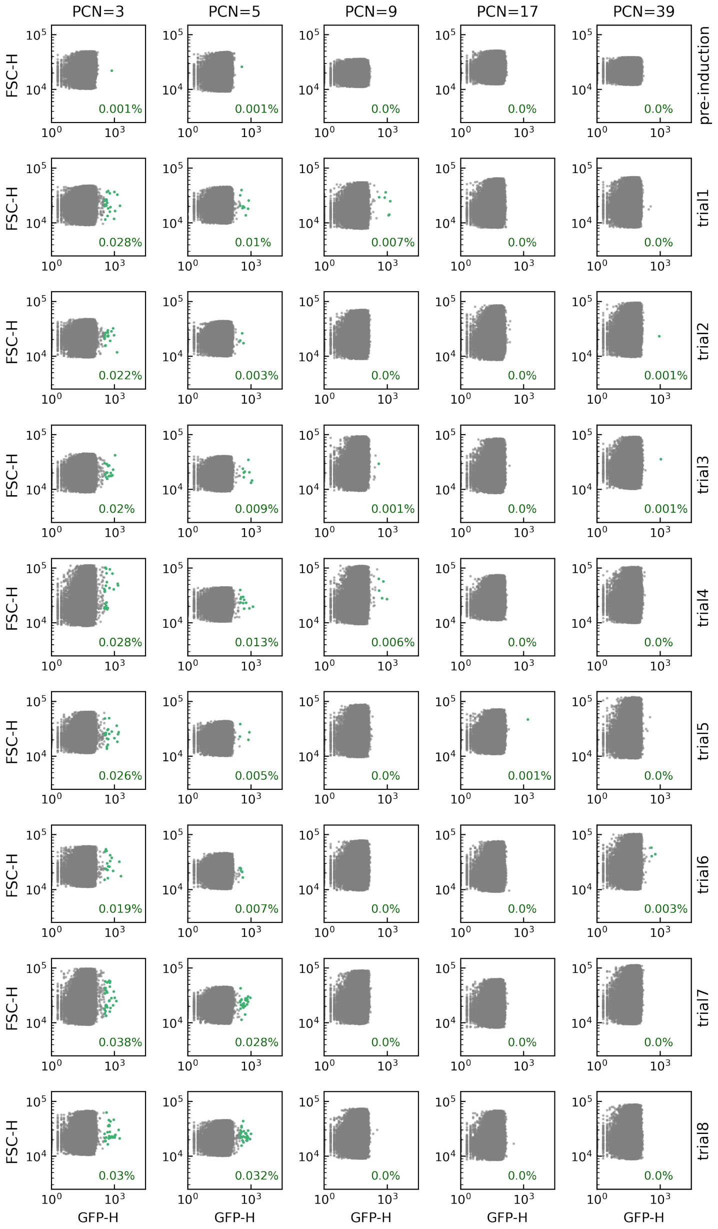

Supplementary Fig. 21. **Flow cytometer data for the circuit in Fig. 4a.** Green dots denote identified mutant events. Rows represent pre-induced culture and independent trials of post-induced cultures. Mutant ratios, labeled in green shown for each trial, correspond to the values in Supplementary Fig. 14. Pre-induced cultures with mutant ratios exceeding 0.01% were excluded from downstream procedures.

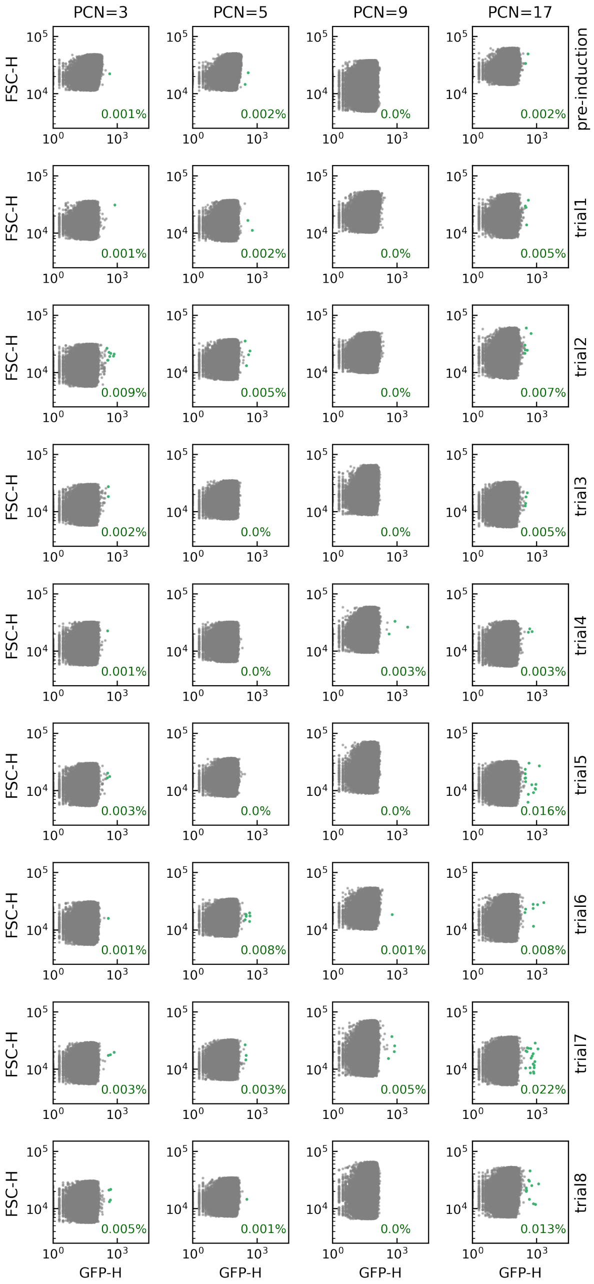

Supplementary Fig. 22. **Flow cytometer data for the circuit in Fig. 6b (sgRNA targets the promoter region).** Green dots denote identified mutant events. Rows represent pre-induced culture and independent trials of post-induced cultures. Mutant ratios, labeled in green shown for each trial, correspond to the values in Supplementary Fig. 15. Pre-induced cultures with mutant ratios exceeding 0.01% were excluded from downstream procedures.

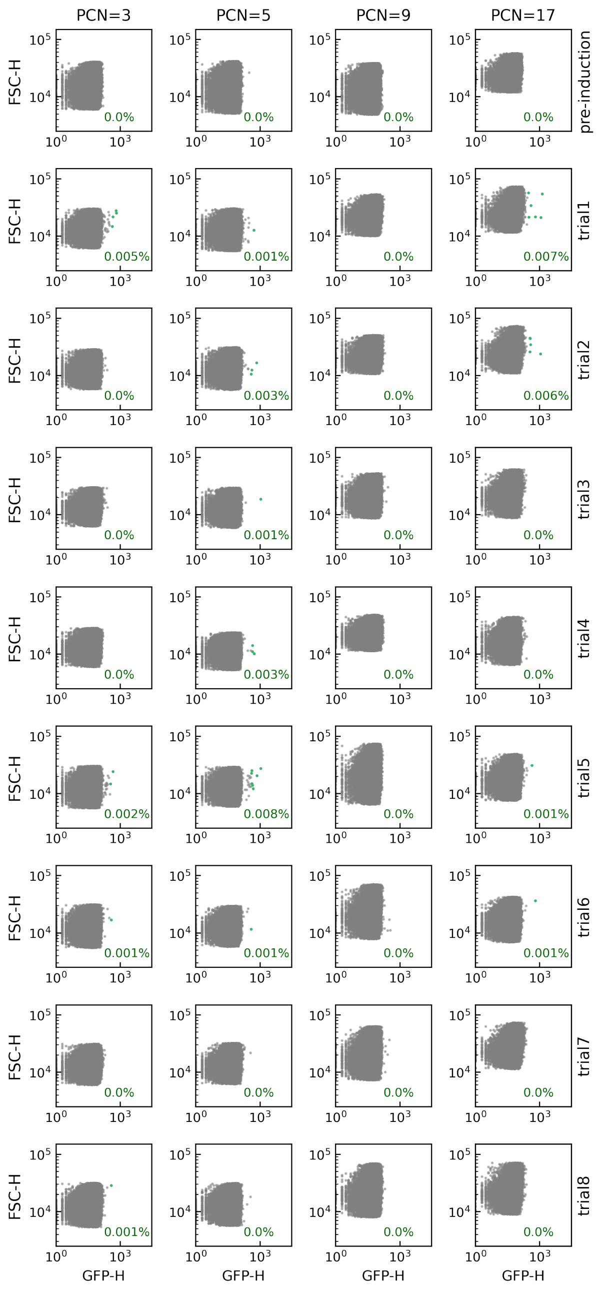

Supplementary Fig. 23. **Flow cytometer data for the circuit in Fig. 6b (sgRNA targets the coding region).** Green dots denote identified mutant events. Rows represent pre-induced culture and independent trials of post-induced cultures. Mutant ratios, labeled in green shown for each trial, correspond to the values in Supplementary Fig. 16. Pre-induced cultures with mutant ratios exceeding 0.01% were excluded from downstream procedures.

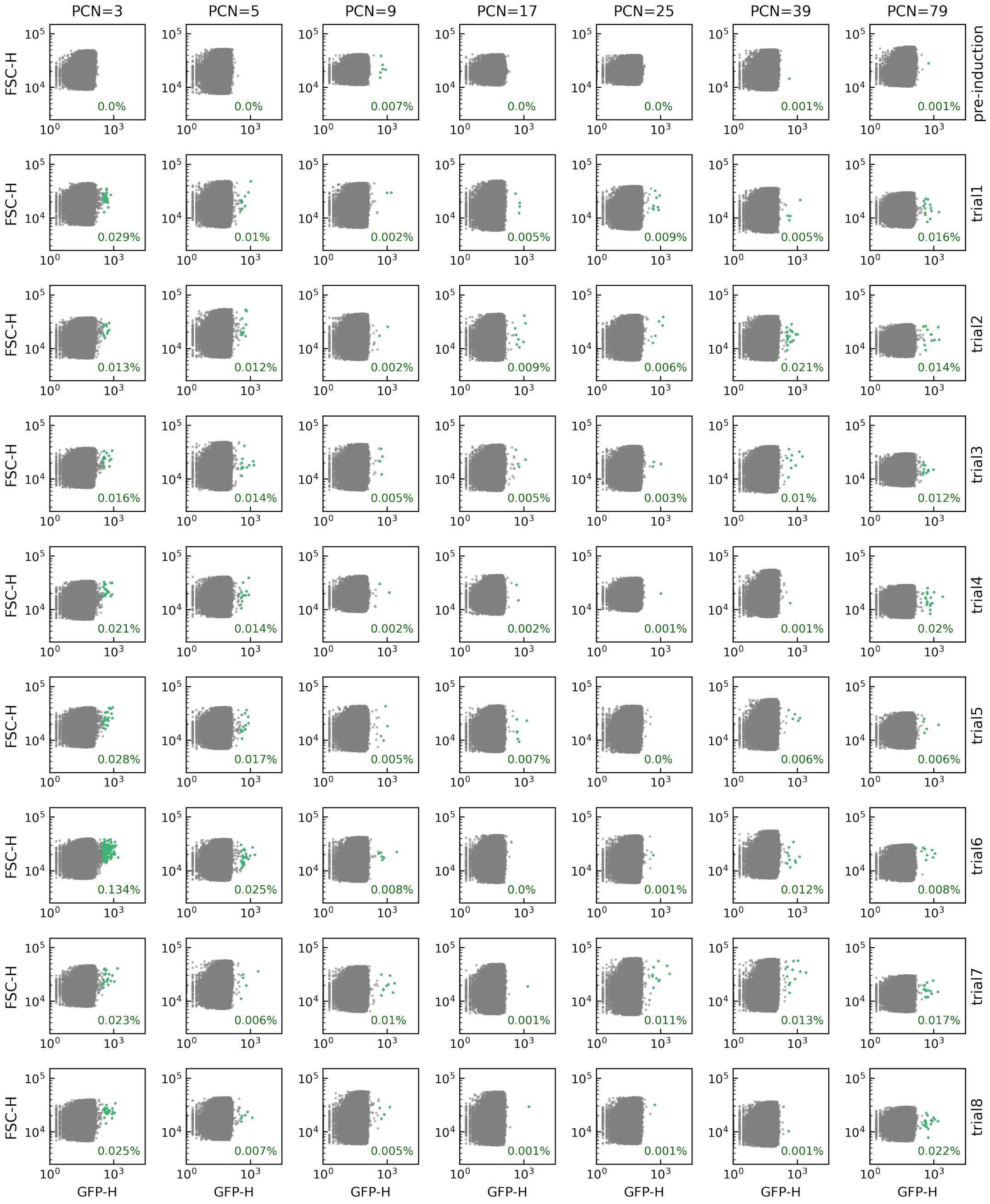

Supplementary Fig. 24. **Flow cytometer data for the circuit in Fig. 6c (sgRNA targets the promoter region).** Green dots denote identified mutant events. Rows represent pre-induced culture and independent trials of post-induced cultures. Mutant ratios, labeled in green shown for each trial, correspond to the values in Supplementary Fig. 17. Pre-induced cultures with mutant ratios exceeding 0.01% were excluded from downstream procedures.

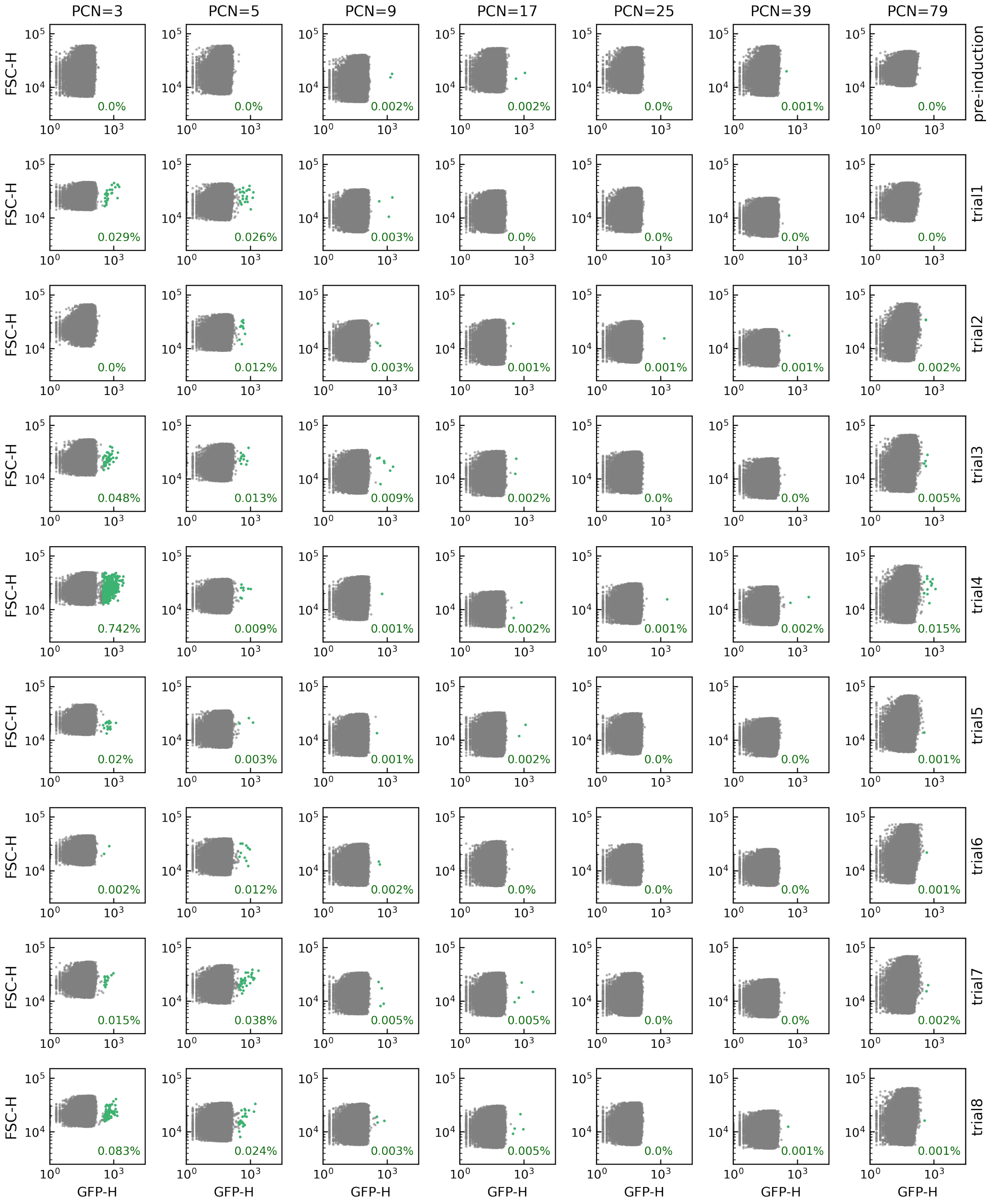

Supplementary Fig. 25. **Flow cytometer data for the circuit in Fig. 6c (sgRNA targets the coding region).** Green dots denote identified mutant events. Rows represent pre-induced culture and independent trials of post-induced cultures. Mutant ratios, labeled in green shown for each trial, correspond to the values in Supplementary Fig. 18. Pre-induced cultures with mutant ratios exceeding 0.01% were excluded from downstream procedures.

7 Plasmid maps and sequences

In Supplementary Fig. 26 we provide the plasmid maps for all constructs used in Figs. 2–6, together with details on key strains, DNA parts, plasmids, primers, and sgRNA properties in Supplementary Tables 2–6.

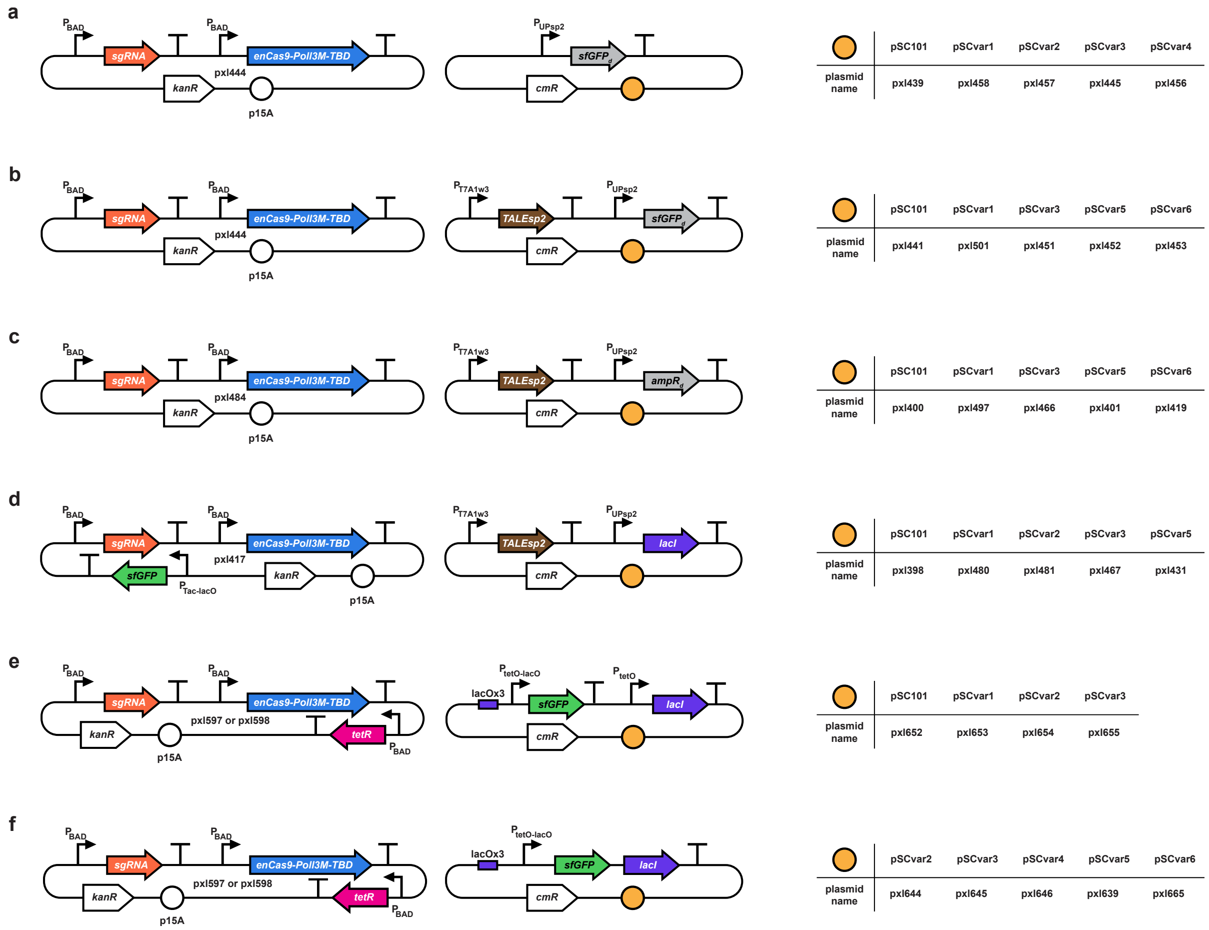

Supplementary Fig. 26. **Plasmid maps of all constructs used.** The corresponding plasmids are listed in Supplementary Table 3. **a** Plasmids used in Fig. 2a. **b** Plasmids used in Fig. 3a. **c** Plasmids used in Fig. 3e. **d** Plasmids used in Fig. 4a. **e** Plasmids used in Fig. 6b (with pxl598 where the sgRNA targets *lacI*) and in Fig. 6c (with pxl597 where the sgRNA targets *lacO*). **f** Plasmids used in Fig. 6b (with pxl598 where the sgRNA targets *lacI*) and in Fig. 6c (with pxl597 where the sgRNA targets *lacO*).

Supplementary Table 2: Bacterial strain genotypes

Strain	Genotype
NEB stable	F' *proA*$^{+}$*B*$^{+}$ *lacI*^{q} *Δ(lacZ)M15 zzf::Tn10 (Tet*^{R}*)/Δ(ara-leu) 7697 araD139 fhuA ΔlacX74 galK16 galE15 e14- Φ80*[illegible] *recA1 relA1 endA1 nupG rpsL (Str*^{R}*) rph spoT1 Δ(mrr-hsdRMS-mcrBC)*
DH5α	*fhuA2Δ(argF-lacZ)U169 phoA glnV44 Φ80Δ(lacZ)M15 gyrA96 recA1 relA1 endA1 thi-1 hsdR17*
DH5αΔlacI::ampR	*ΔlacI::ampR fhuA2Δ(argF-lacZ)U169 phoA glnV44 Φ80Δ(lacZ)M15 gyrA96 recA1 relA1 endA1 thi-1 hsdR17*

Supplementary Table 3: DNA sequence parts

Part	Sequence	Source and comments
EvolvR (enCas9-PolI3M-TBD)	atggacaagaagtattctatcggactggctatcgggactaatagcgtcgggtgggccgt<br>gatcactgacgagtacaaggtgccctctaagaagttcaaggtgctcgggaacaccgacc<br>ggcattccatcaagaaaaatctgatcggagctctcctctttgattcaggggaaaccgct<br>gaagcaacccgcctcaagcggactgctagacggcggtacaccaggaggaagaaccggat<br>ttgttaccttcaagagatattctccaacgaaatggcaaaggtcgacgacagcttcttcc<br>ataggctggaagaatcattcctcgtggaagaggataagaagcatgaacggcatcccatc<br>ttcggtaatatcgtcgacgaggtggcctatcacgagaaatacccaaccatctaccatct<br>tcgcaaaaagctggtggactcaaccgacaaggcagacctccggcttatctacctggccc<br>tggcccacatgatcaagttcagaggccacttcctgatcgagggcgacctcaatcctgac<br>aatagcgatgtggataaactgttcatccagctggtgcagacttacaaccagctctttga<br>agagaaccccatcaatgcaagcggagtcgatgccaaggccattctgtcagcccggctgt<br>caaagagccgcagacttgagaatcttatcgctcagctgccgggtgaaaagaaaaatgga<br>ctgttcgggaacctgattgctctttcacttgggctgactcccaatttcaagtctaattt<br>cgacctggcagaggatgccaagctgcaactgtccaaggacacctatgatgacgatctcg<br>acaacctcctggcccagatcggtgaccaatacgccgaccttttccttgctgctaagaat<br>ctttctgacgccatcctgctgtctgacattctccgcgtgaacactgaaatcaccaaggc<br>ccctctttcagcttcaatgattaagcggtatgatgagcaccaccaggacctgaccctgc<br>ttaaggcactcgtccggcagcagcttccggagaagtacaaggaaatcttctttgaccag<br>tcaaagaatggatacgccggctacatcgacggaggtgcctcccaagaggaattttataa<br>gtttatcaaacctatccttgagaagatggacggcaccgaagagctcctcgtgaaactga<br>atcgggaggatctgctgcggaagcagcgcactttcgacaatgggagcattccccaccag<br>atccatcttggggagcttcacgccatccttcggcgccaagaggacttctacccctttct<br>taaggacaacagggagaagattgagaaaattctcactttccgcatcccctactacgtgg<br>gacccctcgccagaggaaatagccggtttgcttggatgaccagaaagtcagaagaaact<br>atcactccctggaacttcgaagaggtggtggacaagggagccagcgctcagtcattcat<br>cgaacggatgactaacttcgataagaacctccccaatgagaaggtcctgccgaaacatt<br>ccctgctctacgagtactttaccgtgtacaacgagctgaccaaggtgaaatatgtcacc<br>gaagggatgaggaagcccgcattcctgtcaggcgaacaaaagaaggcaattgtggacct<br>tctgttcaagaccaatagaaaggtgaccgtgaagcagctgaaggaggactatttcaaga<br>aaattgaatgcttcgactctgtggagattagcggggtcgaagatcggttcaacgcaagc<br>ctgggtacttaccatgatctgcttaagatcatcaaggacaaggattttctggacaatga<br>ggagaacgaggacatccttgaggacattgtcctgactctcactctgttcgaggaccggg<br>aaatgatcgaggagaggcttaagacctacgcccatctgttcgacgataaagtgatgaag<br>caacttaaacggagaagatataccggatggggacgccttagccgcaaactcatcaacgg<br>aatccgggacaaacagagcggaaagaccattcttgatttccttaagagcgacggattcg<br>ctaatcgcaacttcatgcaacttatccatgatgattccctgacctttaaggaggacatc<br>cagaaggcccaagtgtctggacaaggtgactcactgcacgagcatatcgcaaatctggc<br>tggttcacccgctattaagaagggtattctccagaccgtgaaagtcgtggacgagctgg<br>tcaaggtgatgggtcgccataaaccagagaacattgtcatcgagatggccagggaaaac<br>cagactacccagaagggacagaagaacagcagggagcggatgaaaagaattgaggaagg<br>gattaaggagctcgggtcacagatccttaaagagcacccggtggaaaacacccagcttc<br>agaatgagaagctctatctgtactaccttcaaaatggacgcgatatgtatgtggaccaa<br>gagcttgatatcaacaggctctcagactacgacgtggaccatatcgtccctcagagctt<br>cctcgcagacgactcaattgacaataaggtgctgactcgctcagacaagaaccggggaa<br>agtcagataacgtgccctcagaggaagtcgtgaaaaagatgaagaactattggcgccag<br>cttctgaacgcaaagctgatcactcagcggaagttcgacaatctcactaaggctgagag<br>gggcggactgagcgaactggacaaagcaggattcattaaacggcaacttgtggagactc<br>ggcagattactaaacatgtcgcccaaatccttgactcacgcatgaataccaagtacgac<br>gaaaacgacaaacttatccgcgaggtgaaggtgattaccctgaagtccaagctggtcag<br>cgatttcagaaaggactttcaattctacaaagtgcgggagatcaataactatcatcatg<br>ctcatgacgcatatctgaatgccgtggtgggaaccgccctgatcaagaagtacccagca<br>ctggaaagcgagttcgtgtacggagactacaaggtctacgacgtgcgcaagatgattgc<br>caaatctgagcaggagatcggaaaggccaccgcaaagtacttcttctacagcaacatca<br>tgaatttcttcaagaccgaaatcacccttgcaaacggtgagatccggaaggcgccgctc<br>atcgagactaatggggagactggcgaaatcgtgtgggacaagggcagagatttcgctac<br>cgtgcgcaaagtgctttctatgcctcaagtgaacatcgtgaagaaaaccgaggtgcaaa<br>ccggaggcttttctaaggaatcaatcctccccaagcgcaactccgacaagctcattgca<br>aggaagaaggattgggaccctaagaagtacggcggattcgattcaccaactgtggctta	Halperin *et al*. 2018 Composition: enCas9-linker-PolI3M-TBD.

ttctgtcctggtcgtggctaaggtggaaaaaggaaagtctaagaagctcaagagcgtga
aggaactgctgggtatcaccattatggagcgcagctccttcgagaagaacccaattgac
tttctcgaagccaaaggttacaaggaagtcaagaaggaccttatcatcaagctcccaaa
gtatagcctgttcgaactggagaatgggcggaagcggatgctcgcctccgctggcgaac
ttcagaagggtaatgagctggctctcccctccaagtacgtgaatttcctctaccttgca
agccattacgagaagctgaaggggagccccgaggacaacgagcaaaagcaactgtttgt
ggagcagcataagcattatctggacgagatcattgagcagatttccgagttttctaaac
gcgtcattctcgctgatgccaacctcgataaagtccttagcgcatacaataagcacaga
gacaaaccaattcgggagcaggctgagaatatcatccacctgttcaccctcaccaatct
tggtgcccctgccgcattcaagtacttcgacaccaccatcgaccggaaacgctatacct
ccaccaaagaagtgctggacgccaccctcatccaccagagcatcaccggactttacgaa
actcggattgacctctcacagctcggaggggatggttctagtgaaaccccgggaacaag
tgagtcggccacccctgaaggtggatcaggggggtagcggatccgttcagatcccgcaga
acccgctgattctggttgacggatctagttacctgtaccgtgcttaccatgctttcccg
cctttgaccaattctgctggtgaacctacgggagctatgtacggagttctgaatatgtt
gcgttctttaattatgcagtacaagcctacccacgctgctgttgttttcgatgctaaag
gtaagacgttccgcgacgagttattcgagcactataagtctcaccgtcctccgatgcct
gatgacttacgcgctcagattgagccgctgcatgctatggtgaaggctatgggtttacc
tcttttggctgtcagcggtgttgaggctgatgatgtcattggcaccttagctcgtgagg
ctgagaaggctggtcgccctgttttgatttctaccggtgacaaggacatggctcaattg
gttaccccgaacatcaccctgatcaacaccatgaccaacacgattctgggtcctgagga
agttgttaacaaatatggtgttcctccggagttgattattgactttcttgctcttatgg
gcgattcttcagacaatatcccgggtgttccaggtgttggagagaagactgctcaagct
ctgcttcagggtctgggtggtttggacaccctttacgctgaaccggagaagatcgccgg
tctgtcttttcgcggtgctaagaccatggctgctaaactggaacagaataaggaggtcg
catacctgtcttatcaattggctaccatcaagacggatgtggagttagaacttacgtgc
gagcagcttgaggttcaacagcctgctgctgaggaactgctgggtctttttaagaaata
cgaatttaagcgttggaccgccgacgttgaggctggtaagtggctgcaagctaagggtg
ctaagccggctgctaaaccgcaagaaacgagtgtcgctgatgaggctccggaggttacc
gctaccgttatctcttacgataattatgttacgattctggacgaggaaaccttaaaggc
ttggatcgctaaattagagaaggctcctgttttcgctttcgacacggaaacggattctc
tggacaatattagtgcgaatcttgttggtctgagtttcgcaattgaaccgggtgttgct
gcttacatccctgtggcacacgactacctggacgctccggaccagatttcacgtgaacg
cgctctggaactgctgaagcctttattagaggacgagaaagctttgaaagttggtcaga
atttgaagtatgctcgtggaatcttagctaattatggtatcgagttgcgcggtatcgct
ttcgacacgatgttggaatcttatatcctgaactctgtcgctggtcgccatgacatgga
ctctctggctgagcgctggctgaaacataagacgattaccttcgaggaaatcgcaggaa
agggtaagaaccagctcacgttcaatcaaatcgctctggaggaagctggtcgctatgct
gctgaggacgctgacgttactctgcaactgcacttgaagatgtggcctgacttgcagaa
gcataagggtccactgaatgtttttgaaaacattgagatgcctttggttccagttctgt
ctcgtatcgagcgcaatggcgttaaaattgacccaaaggttttacataaccactcagag
gaactgacgctgcgcttagccgaattggagaaaaaggctaccgaaacgttcggctcgtg
gtatcagcctaaaggtggcactgagatgttctgccatccgcgaacaggtaagccactac
ctaaataccctcgcattaagacacctaaagttggtggtatctttaagaagcctaagaac
aaggcacagcgagaaggccgtgagccttgcgaacttgatacccgcgagtacgttgctgg
tgctccttacaccccagttgaacatgttgtgttcaatctgtcatctacgaaacaactgc
agactatcctgttcgagaagcaaggtatcaagccattaaaaaagacccctggcggtgct
ccgtctacctctgaggaagttttggaggagttagctttggattaccctctgccgaaggt
tatcttggaataccgcggtttggctaaattgaagtctacttatacggataaacttcctt
tgatgattaatccaaagacgggtcgcgttcacacgtcgtaccatcaagctgttaccgct
accggtcgcctgtcttctacggatccgaatttacagaatattcctgtgcgcaatgagga
gggccgccgcattcgtcaagcttttatcgctccggaagactacgttatcgtttctgctg
attattctcaaaatgaattacgtatcatggctcacctgtctcgcgataagggtctgttg
acggcctttgctgagggtaaggacattcatcgtgctaccgctgctgaggttttcggcct
gccgttggaaacggttacgtctgaacagcgtcgctctgctaagcgtattaatttcggct
taatctacggtatgtctgcgtttggcttagctcgtcagctgaatatcccgcgcaaggaa
gctcaaaaatatatggatctgtattttgagcgttacccgggtgttttggaatacatgga
gcgtacgcgcgcgcaagctaaggaacaaggttatgtggaaaccttggatggtcgtcgct
tgtacttgcctgacattaagtcttctaacggcgcccgccgcgctgctgccgagcgcgct
gctatcaatgctccgatgcaaggtactgctgctgatattattaagcgtgctatgatcgc
tgtggacgcttggctgcaagctgaacagcctcgcgttcgcatgattatgcaagttcatg
acgagttggttttcgaggtgcataaggacgacgtggacgctgttgctaaacaaatccac
cagttgatggagaattgcacgcgcttagacgttccgctgctggttgaagttggttctgg
tgaaaactgggaccaggctcactaa

P_{BAD}

agaaaccaattgtccatattgcatcagacattgccgtcactgcgtcttttactggctc
ttctcgctaaccaaaccggtaaccccgcttattaaaagcattctgtaacaaagcggga
ccaaagccatgacaaaaacgcgtaacaaaagtgtctataatcacggcagaaaagtcca
cattgattatttgcacggcgtcacactttgctatgccatagcatttttatccataaga
ttagcggatcctac**ctgacg**cttttatcgcaactc**tctact**gtttctccat

Meyer *et al*. 2018
-35, -10

sgRNA targeting sfGFP$_{d}$	acccgacatctaaccggaaggctatgtgcgttttagagctagaaatagcaagttaaaa<br>taaggctagtccgttatcaacttgaaaaagtggcaccgagtcggtgcttttttt	targeting region<br>sgRNA scaffold
sgRNA targeting ampR$_{d}$	aacctgataagataacactggttttagagctagaaatagcaagttaaaataaggctag<br>acctaactcaacttggacttcggtccaagtggcaccgagtcggtgcttttttt	Riesenberg *et al*. 2022<br>targeting region<br>sgRNA scaffold with hairpin
sgRNA targeting lacI	acccggctagccgtttcccgcgtggtgaaccgttttagagctagaaatagcaagttaa<br>aataaggctagtccgttatcaacttgaaaaagtggcaccgagtcggtgcttttttt	targeting region<br>sgRNA scaffold
sgRNA targeting lacO	ctcacaattggtggagcgctgttttagagctagaaatagcaagttaaaataaggctag<br>tccgttatcaacttggacttcggtccaagtggcaccgagtcggtgcttttttt	targeting region<br>sgRNA scaffold with hairpin
$P_{Tac-lacO}$	**ttgaca**attaatcatcggctcg**tataat**gtgtggaattgtgagcgctcacaatt	Meyer *et al*. 2018<br>lacO operator **-35, -10**
sfGFP$_{1}$	atgcgtaaaggcgaagagctgttcactggtgtcgtccctattctggtggaactggatg<br>gtgatgtcaacggtcataagttttccgtgcgtggcgagggtgaaggtgacgcaactaa<br>tggtaaactgacgctgaagttcatctgtactactggtaaactgccggtaccttggccg<br>actctggtaacgacgctgacttatggtgttcagtgctttgctcgttatccggaccata<br>tgaagcagcatgacttcttcaagtccgccatgccggaaggctatgtgcaggaacgcac<br>gatttcctttaaggatgacggcacgtacaaaacgcgtgcggaagtgaaatttgaaggc<br>gataccctggtaaaccgcattgagctgaaaggcattgactttaaagaagacggcaata<br>tcctgggccataagctggaatacaattttaacagccacaatgtttacatcaccgccga<br>taaacaaaaaaatggcattaaagcgaattttaaaattcgccacaacgtggaggatggc<br>agcgtgcagctggctgatcactaccagcaaaacactccaatcggtgatggtcctgttc<br>tgctgccagacaatcactatctgagcacgcaaagcgttctgtctaaagatccgaacga<br>gaaacgcgatcatatggttctgctggagttcgtaaccgcagcgggcatcacgcatggt<br>atggatgaactgtacaaatga	Daeffler *et al*. 2017
P_{UPsp2}	atcccgaaaatttatcaaaaagagtattgacttatattgagtcgtataggatacttac<br>agccatcgagagctgcg	Segall-Shapiro *et al.* 2018
P_{T7A1w3}	ggcgcgcctcagtcagagtattgacttaaagtctaacctataggagatctacagccat<br>cgagagctgcg	Segall-Shapiro *et al.* 2018
TALEsp2	atggtagatttaagaactttaggatattcacagcagcaacaggaaaagatcaagccca<br>aagttaggtcgacagtcgcgcagcatcacgaagcgctggttggtcatgggtttacaca<br>tgcccacatcgtagccttatcgcagcaccctgcagcccttggcacggtcgccgtcaag<br>taccaggacatgattgcggcgttgccggaagccacacatgaggcgatcgtcggtgtgg<br>ggaaacagtggagcggagcccgagcgcttgaggccctgttgacggtcgcgggagagct<br>gagagggcctccccttcagctggacacgggccagttgctgaagatcgcgaagcgggga<br>ggagtcacggcggtcgaggcggtgcacgcgtggcgcaatgcgctcacgggagcacccc<br>tcaacctgaccccggaccaggtagtcgcgattgcttcacatgacggggggtaaacaagc<br>gctggaaacggtgcagcgtctgctaccggtgttatgtcaggatcatgggctcacgccg<br>gaacaggtagtggcaattgcgagtcatgacggtggcaaacaggccctggaaaccgtac<br>agcggctgctaccggtgctgtgtcaagcgcatggcctgactccggaccaagttgtagc<br>cattgcctcgaacgggggcggcaagcaggcgctggagactgttcaacgtctgctcccc<br>gttctgtgtcaggcgcatggcctgacgcctgcgcaggtcgtggcgatcgcttcaaaca<br>tcggtgggaagcaagccctggagactgtccaaagactgttgccagtgttgtgtcaaga<br>tcatggcttaaccccagatcaggtggttgcgattgcatcaaatggaggtggtaaacag<br>gcgctggagactgtgcagcgcctgttgccggttctgtgccaagatcatgggctgactc<br>cggaacaggttgtggctatcgcaagcaatattggtggcaagcaggccctggaaacagt<br>acagcgcctgctgcctgtattgtgtcaagcccacggtcttaccccccgatcaggtagtc<br>gccatagcatcgcacgacggcgggaagcaggcccttgagactgtacaacgcctcctgc<br>cggttttgtgccaggcgcacggcctgacgccagcccaggtggttgcgatagccagtaa<br>taacggcggtaagcaagcccttgaaaccgttcaacgtttgctgccagtgctgtgccag<br>gatcacggcctgaccccggatcaggtagtcgccattgctagcaacattggtggcaaac<br>aagcactggagacagttcaacgcttactgcccgtgctttgccaggatcatggactgac<br>cccagagcaagtggtcgcgattgcctcgcatgacggaggtaaacaggcccttgagact<br>gtccagcgtctgctgccggtcctttgccaggctcatgggctgacgcccgaccaggtgg<br>tagcaatcgcttcgaacggcggaggcaaacaggcgttagaaaccgttcaacgtctgtt<br>accggtgctgtgccaagctcatggcttaaccccggcccaggtagtcgcaatcgctagt<br>catgatggtgggaaacaggcattagaaacagtacaacgtctgctcccggtcctctgtc<br>aggatcacggtctgaccccggatcaggttgtagcaatcgctagcaatattggtggtaa<br>acaggcgctggaaacagtgcaaagattattaccagttctgtgtcaggaccatggcctg<br>acacctgagcaggtcgtagcgatcgcaagtaatattggaggtaaacaggccctggaaa<br>ccgtgcagcgtctgctgcctgtgctctgtcaagcgcatggtctgactccggatcaggt<br>tgtcgcgatcgccagtaacgggggagggaaacaggcactcgagactgtgcagagattg	Segall-Shapiro *et al.* 2018

ctgccggtcctgtgtcaggcgcatggtctgaccccagcgcaggtcgtggcaatcgctt
ccaacataggtggtaaacaggccctcgaaactgtccaacggctgttaccggtactgtg
ccaggatcatggtctgacccctgagcaggtagtggctattgcatccaacggagggggc
agacccgcactggagtcaatcgtggcccagctttcgaggccggaccccgcgctggccg
cactcactaatgatcatcttgtagcgctggcctgcctcggcggacgacccgccttgga
tgcggtgaagaaggggctcccgcacgcgcctgcattgattaagcggaccaacagaagg
attcccgagaggacatcacatcgagtggcagatcacgcgcaagtggtccgcgtgctcg
gattcttccagtgtcactcccaccccgcacaagcgttcgatgacgccatgactcaatt
tggtatgtcgagacacggactgctgcagctctttcgtagagtcggtgtcacagaactc
gaggcccgctcgggcacactgcctcccgcctcccagcggtgggacaggattctccaag
cgagcggtatgaaacgcgcgaagccttcacctacgtcaactcagacacctgaccaggc
gagccttcatgcgttcgcagactcgctggagagggatttggacgcgccctcgcccatg
catgaaggggaccaaactcgcgcgtcataa

pSC101

gagttatacacagggctgggatctattctttttatctttttttattctttctttattc
tataaattataaccacttgaatataaacaaaaaaaacacacaaaggtctagcggaatt
tacagagggtctagcagaatttacaagttttccagcaaaggtctagcagaatttacag
atacccacaactcaaaggaaaaggactagtaattatcattgactagcccatctcaatt
ggtatagtgattaaaatcacctagaccaattgagatgtatgtctgaattagttgtttt
caaagcaaatgaactagcgattagtcgctatgacttaacggagcatgaaaccaagcta
attttatgctgtgtggcactactcaaccccacgattgaaaaccctacaaggaaagaac
ggacggtatcgttcacttataaccaatacgctcagatgatgaacatcagtagggaaaa
tgcttatggtgtattagctaaagcaaccagagagctgatgacgagaactgtggaaatc
aggaatcctttggttaaaggctttgagattttccagtggacaaactatgccaagttct
caagcgaaaaattagaattagtttttagtgaagagatattgccttatcttttccagtt
aaaaaaattcataaaatataatctggaacatgttaagtcttttgaaaacaaatactct
atgaggatttatgagtggttattaaaagaactaacacaaaagaaaactcacaaggcaa
atatagagattagccttgatgaatttaagttcatgttaatgcttgaaaataactacca
tgagtttaaaaggcttaaccaatgggttttgaaaccaataagtaaagatttaaacact
tacagcaatatgaaattggtggttgataagcgaggccgcccgactgatacgttgattt
tccaagttgaactagatagacaaatggatctcgtaaccgaacttgagaacaaccagat
aaaaatgaatggtgacaaaataccaacaaccattacatcagattcctacctacataac
ggactaagaaaaacactacacgatgctttaactgcaaaaattcagctcaccagttttg
aggcaaaatttttgagtgacatgcaaagtaagtatgatctcaatggttcgttctcatg
gctcacgcaaaaacaacgaaccacactagagaacatactggctaaatacggaaggatc
tga

Segall-Shapiro *et al.* 2018

pSC101 variant1

gagttatacacagggctgggatctattctttttatctttttttattctttctttattc
tataaattataaccacttgaatataaacaaaaaaaacacacaaaggtctagcggaatt
tacagagggtctagcagaatttacaagttttccagcaaaggtctagcagaatttacag
atacccacaactcaaaggaaaaggactagtaattatcattgactagcccatctcaatt
ggtatagtgattaaaatcacctagaccaattgagatgtatgtctgaattagttgtttt
caaagcaaatgaactagcgattagtcgctatgacttaacggagcatgaaaccaagcta
attttatgctgtgtggcactactcaaccccacgattgaaaaccctacaaggaaagaac
ggacggtatcgttcacttataaccaatacgctcagatgatgaacatcagtagggaaaa
tgcttatggtgtattagctaaagcaaccagagagctgatgacgagaactgtggaaatc
aggaatcctttggttaaaggctttg**tt**attttccagtggacaaactatgccaagttct
caagcgaaaaattagaattagtttttagtgaagagatattgccttatcttttccagtt
aaaaaaattcataaaatataatctggaacatgttaagtcttttgaaaacaaatactct
atgaggatttatgagtggttattaaaagaactaacacaaaagaaaactcacaaggcaa
atatagagattagccttgatgaatttaagttcatgttaatgcttgaaaataactacca
tgagtttaaaaggcttaaccaatgggttttgaaaccaataagtaaagatttaaacact
tacagcaatatgaaattggtggttgataagcgaggccgcccgactgatacgttgattt
tccaagttgaactagatagacaaatggatctcgtaaccgaacttgagaacaaccagat
aaaaatgaatggtgacaaaataccaacaaccattacatcagattcctacctacataac
ggactaagaaaaacactacacgatgctttaactgcaaaaattcagctcaccagttttg
aggcaaaatttttgagtgacatgcaaagtaagtatgatctcaatggttcgttctcatg
gctcacgcaaaaacaacgaaccacactagagaacatactggctaaatacggaaggatc
tga

Segall-Shapiro *et al.* 2018
mutation relative to pSC101

pSC101 variant2

gagttatacacagggctgggatctattctttttatctttttttattctttctttattc
tataaattataaccacttgaatataaacaaaaaaaacacacaaaggtctagcggaatt
tacagagggtctagcagaatttacaagttttccagcaaaggtctagcagaatttacag
atacccacaactcaaaggaaaaggactagtaattatcattgactagcccatctcaatt
ggtatagtgattaaaatcacctagaccaattgagatgtatgtctgaattagttgtttt
caaagcaaatgaactagcgattagtcgctatgacttaacggagcatgaaaccaagcta
attttatgctgtgtggcactactcaaccccacgattgaaaaccctacaaggaaagaac
ggacggtatcgttcacttataaccaatacgctcagatgatgaacatcagtagggaaaa
tgcttatggtgtattagctaaagcaaccagagagctgatgacgagaactgtggaaatc
aggaatcctttggttaaaggcttt**tg**gattttccagtggacaaactatgccaagttct
caagcgaaaaattagaattagtttttagtgaagagatattgccttatcttttccagtt

Segall-Shapiro *et al.* 2018
mutation relative to pSC101

aaaaaaattcataaaatataatctggaacatgttaagtcttttgaaaacaaatactct
atgaggatttatgagtggttattaaaagaactaacacaaaagaaaactcacaaggcaa
atatagagattagccttgatgaatttaagttcatgttaatgcttgaaaataactacca
tgagtttaaaaggcttaaccaatgggttttgaaaccaataagtaaagatttaaacact
tacagcaatatgaaattggtggttgataagcgaggccgcccgactgatacgttgattt
tccaagttgaactagatagacaaatggatctcgtaaccgaacttgagaacaaccagat
aaaaatgaatggtgacaaaataccaacaaccattacatcagattcctacctacataac
ggactaagaaaaacactacacgatgctttaactgcaaaaattcagctcaccagttttg
aggcaaaatttttgagtgacatgcaaagtaagtatgatctcaatggttcgttctcatg
gctcacgcaaaaacaacgaaccacactagagaacatactggctaaatacggaaggatc
tga

pSC101 variant3

gagttatacacagggctgggatctattcttttttatcttttttttattctttctttattc
tataaattataaccacttgaatataaacaaaaaaaacacacaaaggtctagcggaatt
tacagagggtctagcagaatttacaagttttccagcaaaggtctagcagaatttacag
atacccacaactcaaaggaaaaggactagtaattatcattgactagcccatctcaatt
ggtatagtgattaaaatcacctagaccaattgagatgtatgtctgaattagttgtttt
caaagcaaatgaactagcgattagtcgctatgacttaacggagcatgaaaccaagcta
attttatgctgtgtggcactactcaaccccacgattgaaaaccctacaaggaaagaac
ggacggtatcgttcacttataaccaatacgctcagatgatgaacatcagtagggaaaa
tgcttatggtgtattagctaaagcaaccagagagctgatgacgagaactgtggaaatc
aggaatcctttggttaaaggcttt**a**agattttccagtggacaaactatgccaagttct
caagcgaaaaaattagaattagtttttagtgaagagatattgccttatcttttccagtt
aaaaaaattcataaaatataatctggaacatgttaagtcttttgaaaacaaatactct
atgaggatttatgagtggttattaaaagaactaacacaaaagaaaactcacaaggcaa
atatagagattagccttgatgaatttaagttcatgttaatgcttgaaaataactacca
tgagtttaaaaggcttaaccaatgggttttgaaaccaataagtaaagatttaaacact
tacagcaatatgaaattggtggttgataagcgaggccgcccgactgatacgttgattt
tccaagttgaactagatagacaaatggatctcgtaaccgaacttgagaacaaccagat
aaaaatgaatggtgacaaaataccaacaaccattacatcagattcctacctacataac
ggactaagaaaaacactacacgatgctttaactgcaaaaattcagctcaccagttttg
aggcaaaatttttgagtgacatgcaaagtaagtatgatctcaatggttcgttctcatg
gctcacgcaaaaacaacgaaccacactagagaacatactggctaaatacggaaggatc
tga

Segall-Shapiro *et al.* 2018
mutation relative to pSC101

pSC101 variant4

gagttatacacagggctgggatctattcttttttatcttttttttattctttctttattc
tataaattataaccacttgaatataaacaaaaaaaacacacaaaggtctagcggaatt
tacagagggtctagcagaatttacaagttttccagcaaaggtctagcagaatttacag
atacccacaactcaaaggaaaaggactagtaattatcattgactagcccatctcaatt
ggtatagtgattaaaatcacctagaccaattgagatgtatgtctgaattagttgtttt
caaagcaaatgaactagcgattagtcgctatgacttaacggagcatgaaaccaagcta
attttatgctgtgtggcactactcaaccccacgattgaaaaccctacaaggaaagaac
ggacggtatcgttcacttataaccaatacgctcagatgatgaacatcagtagggaaaa
tgcttatggtgtattagctaaagcaaccagagagctgatgacgagaactgtggaaatc
aggaatcctttggttaaaggctttg**g**gattttccagtggacaaactatgccaagttct
caagcgaaaaaattagaattagtttttagtgaagagatattgccttatcttttccagtt
aaaaaaattcataaaatataatctggaacatgttaagtcttttgaaaacaaatactct
atgaggatttatgagtggttattaaaagaactaacacaaaagaaaactcacaaggcaa
atatagagattagccttgatgaatttaagttcatgttaatgcttgaaaataactacca
tgagtttaaaaggcttaaccaatgggttttgaaaccaataagtaaagatttaaacact
tacagcaatatgaaattggtggttgataagcgaggccgcccgactgatacgttgattt
tccaagttgaactagatagacaaatggatctcgtaaccgaacttgagaacaaccagat
aaaaatgaatggtgacaaaataccaacaaccattacatcagattcctacctacataac
ggactaagaaaaacactacacgatgctttaactgcaaaaattcagctcaccagttttg
aggcaaaatttttgagtgacatgcaaagtaagtatgatctcaatggttcgttctcatg
gctcacgcaaaaacaacgaaccacactagagaacatactggctaaatacggaaggatc
tga

Segall-Shapiro *et al.* 2018
mutation relative to pSC101

pSC101 variant5

gagttatacacagggctgggatctattcttttttatcttttttttattctttctttattc
tataaattataaccacttgaatataaacaaaaaaaacacacaaaggtctagcggaatt
tacagagggtctagcagaatttacaagttttccagcaaaggtctagcagaatttacag
atacccacaactcaaaggaaaaggactagtaattatcattgactagcccatctcaatt
ggtatagtgattaaaatcacctagaccaattgagatgtatgtctgaattagttgtttt
caaagcaaatgaactagcgattagtcgctatgacttaacggagcatgaaaccaagcta
attttatgctgtgtggcactactcaaccccacgattgaaaaccctaca**t**ggaaagaac
ggacggtatcgttcacttataaccaatacgctcagatgatgaacatcagtagggaaaa
tgcttatggtgtattagctaaagcaaccagagagctgatgacgagaactgtggaaatc
aggaatcctttggttaaaggctttgagattttccagtggacaaactatgccaagttct
caagcgaaaaaattagaattagtttttagtgaagagatattgccttatcttttccagtt
aaaaaaattcataaaatataatctggaacatgttaagtcttttgaaaacaaatactct
atgaggatttatgagtggttattaaaagaactaacacaaaagaaaactcacaaggcaa
atatagagattagccttgatgaatttaagttcatgttaatgcttgaaaataactacca

Segall-Shapiro *et al.* 2018
mutation relative to pSC101

	tgagtttaaaaggcttaaccaatgggttttgaaaccaataagtaaagatttaaacact<br>tacagcaatatgaaattggtggttgataagcgaggccgcccgactgatacgttgattt<br>tccaagttgaactagatagacaaatggatctcgtaaccgaacttgagaacaaccagat<br>aaaaatgaatggtgacaaaataccaacaaccattacatcagattcctacctacataac<br>ggactaagaaaaacactacacgatgctttaactgcaaaaattcagctcaccagttttg<br>aggcaaaatttttgagtgacatgcaaagtaagtatgatctcaatggttcgttctcatg<br>gctcacgcaaaaacaacgaa**t**cacactagagaacatactggctaaatacggaaggatc<br>tga	
pSC101 variant6	gagttatacacagggctgggatctattctttttatcttttttttattctttctttattc<br>tataaattataaccacttgaatataaacaaaaaaaacacacaaaggtctagcggaatt<br>tacagagggtctagcagaatttacaagttttccagcaaaggtctagcagaatttacag<br>atacccacaactcaaaggaaaaggactagtaattatcattgactagcccatctcaatt<br>ggtatagtgattaaaatcacctagaccaattgagatgtatgtctgaattagttgtttt<br>caaagcaaatgaactagcgattagtcgctatgacttaacggagcatgaaaccaagcta<br>attttatgctgtgtggcactactcaaccccacgattgaaaaccctacaaggaaagaac<br>ggacggtatcgttcacttataaccaatacgctcagatgatgaacatcagtagggaaaa<br>tgcttatggtgtattagctaaagcaaccagagagctgatgacgagaactgtggaaatc<br>aggaatcctttggttaaaggctttgagattttccagtggaca**g**actatgccaagttct<br>caagcgaaaaattagaattagtttttagtgaagagatattgccttatcttttccagtt<br>aaaaaaattcataaaatataatctggaacatgttaagtcttttgaaaacaaatactct<br>atgaggatttatgagtggttattaaaagaactaacacaaaagaaaactcacaaggcaa<br>atatagagattagccttgatgaatttaagttcatgttaatgcttgaaaataactacca<br>tgagtttaaaaggcttaaccaatgggttttgaaaccaataagtaaagatttaaacact<br>tacagcaatatgaaattggtggttgataagcgaggccgcccgactgatacgttgattt<br>tccaagttgaactagatagacaaatggatctcgtaaccgaacttgagaacaaccagat<br>aaaaatgaatggtgacaaaataccaacaaccattacatcagattcctacctacataac<br>ggactaagaaaaacactacacgatgctttaactgcaaaaattcagctcaccagttttg<br>aggcaaaatttttgagtgacatgcaaagtaagtatgatctcaatggttcgttctcatg<br>gctcacgcaaaaacaacgaaccacactagagaacatactggctaaatacggaaggatc<br>tga	Segall-Shapiro *et al.* 2018<br>**mutation relative to pSC101**
synthetic P_{tetO} promoter	gggctttttttattgcgcaagcttcg**ttgaca**tccctatcagtgatagaga**tataat**gc<br>ac	Meyer *et al*. 2018.<br>tetO operator<br>**-35**, **-10**
$lacO_{decoy3x}$-$P_{tetO-lacO}$ promoter	ttgtgagcggataacaacatgctcgtgacagaattgtgagcgctcacaattactagcg<br>gccgcatggttgtgagcggataacaacgcttaacgatcgttggctgttttcagcagga<br>cgcactgacctccctatcagtgatagaga**ttgaca**tccctatcagtgataga**ga****tac**<br>**t**gagcagaattgtgagcgctcacaattggtggagcgctcggtttttt	Lee *et al*. 2016.<br>sgRNA target<br>lacO operator<br>tetO operator<br>**-35**, **-10**
$sfGFP_d$	atgagcaaaggagaagaacttttcactggagttgtcccaattcttgttgaattagatg<br>gtgatgttaatgggcacaaattttctgtccgtggagagggtgaaggtgatgctacaaa<br>cggaaaactcacccttaaatttatttgcactactggaaaactacctgttccgtggcca<br>acacttgtcactactctgacctatggtgttcaatgcttttcccgttatccggatcaca<br>tgaaacggcatgacttcttcaagtccgcctaaccggaaggctatgtgcaggaacgcac<br>tatatctttcaaagatgacgggacctacaagacgcgtgctgaagtcaagtttgaaggt<br>gatacccttgttaatcgtatcgagttaaagggtattgattttaaagaagatggaaaca<br>ttcttggacacaaactcgagtacaactttaactcacacaatgtatacatcacggcaga<br>caaacaaaagaatggaatcaaagctaacttcaaaattcgccacaacgttgaagatggt<br>tccgttcaactagcagaccattatcaacaaaatactccaattggcgatggccctgtcc<br>ttttaccagacaaccattacctgtcgacacaatctgtcctttcgaaagatcccaacga<br>aaagcgtgaccacatggtccttcttgagtttgtaactgctgctgggattacacatggc<br>atggatgagctctacaaataa	sgRNA target<br>premature stop codon
$ampR_d$	atgagtattcaacatttccgtgtcgcccttattcccttttttgcggcattttgccttc<br>ctgtttttgctcacccagaaacgctggtgaaagtaaaagatgctgaagatcagttggg<br>tgcacgagtgggttacatcgaactggatctcaacagcggtaagatccttgagagtttt<br>cgccccgaagaacgttttccaatgatgagcacttttaaagttctgctatgtggcgcgg<br>tattatcccgtattgacgccgggcaagagcaactcggtcgccgcatacactattctca<br>gaatgacttggttgagtactcaccagtcacagaaaagcatcttacggatggcatgaca<br>gtaagagaattatgcagtgctgccataacctgataagataacactgcggccaacttac<br>ttctgacaacgatcggaggaccgaaggagctaaccgcttttttgcacaacatggggga<br>tcatgtaactcgccttgatcgttgggaaccggagctgaatgaagccataccaaacgac<br>gagcgtgacaccacgatgcctgtagcaatggcaacaacgttgcgcaaactattaactg<br>gcgaactacttactctagcttcccggcaacaattaatagactggatggaggcggataa<br>agttgcaggaccacttctgcgctcggcccttccggctggctggtttattgctgataaa<br>tctggagccggtgagcgtgggtcgcgcggtatcattgcagcactggggccagatggta<br>agccctcccgtatcgtagttatctacacgacggggagtcaggcaactatggatgaacg<br>aaatagacagatcgctgagataggtgcctcactgattaagcattggtaa	sgRNA target<br>premature stop codon

lacI	atgaaaccagtaacgttatacgatgtcgcagagtatgccggtgtctcttatatgaccg<br>tttcccgcgtggtgaaccaggccagccacgtttctgcgaaaacgcgggaaaaagtgga<br>agcggcgatggtggagctgaattacattcccaaccgcgtggcacaacaactggcgggc<br>aaacagtcgttgctgattggcgttgccacctccagtctggccctgcacgcgccgtcgc<br>aaattgtcgcggcgattaaatctcgcgccgatcaactgggtgccagcgtggtggtgtc<br>gatggtagaacgaagcggcgtcgaagcctgtaaagcggcggtgcacaatcttctcgcg<br>caacgcgtcagtgggctgatcattaactatccgctggatgaccaggatgccattgctg<br>tggaagctgcctgcactaatgttccggcgttatttcttgatgtctctgaccagacacc<br>catcaacagtattattttctcccatgaggacggtacgcgactgggcgtggagcatctg<br>gtcgcattgggtcaccagcaaatcgcgctgttagcgggcccattaagttctgtctcgg<br>cgcgtctgcgtctggctggctggcataaatatctcactcgcaatcaaattcagccgat<br>agcggaacgggaaggcgactggagtgccatgtccggttttcaacaaaccatgcaaatg<br>ctgaatgagggcatcgttcccactgcgatgctggttgccaacgatcagatggcgctgg<br>gcgcaatgcgcgccattaccgagtccgggctgcgcgttggtgcggatatctcggtagt<br>gggatacgacgataccgaagatagctcatgttatatcccgccgttaaccaccatcaaa<br>caggattttcgcctgctggggcaaaccagcgtggaccgcttgctgcaactctctcagg<br>gccaggcggtgaagggcaatcagctgttgccagtctcactggtgaaaagaaaaaccac<br>cctggcgcccaatacgcaaaccgcctctccccgcgcgttggccgattcattaatgcag<br>ctggcacgacaggtttcccgactggaaagcgggcaggcggcgaataaaaatgaagaaa<br>ataccaatgaagtgccgacctttatgctgaatgcgggccaggcgaattatgcgtttgc<br>gtaa	Meyer *et al*. 2018<br>sgRNA target
sfGFP$_2$	atgagcaaaggagaagaacttttcactggagttgtcccaattcttgttgaattagatg<br>gtgatgttaatgggcacaaattttctgtccgtggagagggtgaaggtgatgctacaaa<br>cggaaaactcacccttaaatttatttgcactactggaaaactacctgttccgtggcca<br>acacttgtcactactctgacctatggtgttcaatgcttttcccgttatccggatcaca<br>tgaaacggcatgactttttcaagagtgccatgcccgaaggttatgtacaggaacgcac<br>tatatctttcaaagatgacgggacctacaagacgcgtgctgaagtcaagtttgaaggt<br>gatacccttgttaatcgtatcgagttaaagggtattgattttaaagaagatggaaaca<br>ttcttggacacaaactcgagtacaactttaactcacacaatgtatacatcacggcaga<br>caaacaaaagaatggaatcaaagctaacttcaaaattcgccacaacgttgaagatggt<br>tccgttcaactagcagaccattatcaacaaaatactccaattggcgatggccctgtcc<br>ttttaccagacaaccattacctgtcgacacaatctgtcctttcgaaagatcccaacga<br>aaagcgtgaccacatggtccttcttgagtttgtaactgctgctgggattacacatggc<br>atggatgagctctacaaataa	Segall-Shapiro *et al.* 2018
tetR	atgtccagattagataaaagtaaagtgattaacagcgcattagagctgcttaatgagg<br>tcggaatcgaaggtttaacaacccgtaaactcgcccagaagctaggtgtagagcagcc<br>tacattgtattggcatgtaaaaaataagcgggctttgctcgacgccttagccattgag<br>atgttagataggcaccatactcacttttgccctttagaaggggaaagctggcaagatt<br>ttttacgtaataacgctaaaagttttagatgtgctttactaagtcatcgcgatggagc<br>aaaagtacatttaggtacacggcctacagaaaaacagtatgaaactctcgaaaatcaa<br>ttagccttttatgccaacaaggtttttcactagagaatgcattatatgcactcagcg<br>ctgtggggcattttactttaggttgcgtattggaagatcaagagcatcaagtcgctaa<br>agaagaaagggaaacacctactactgatagtatgccgccattattacgacaagctatc<br>gaattatttgatcaccaaggtgcagagccagccttcttattcggccttgaattgatca<br>tatgcggattagaaaaacaacttaaatgtgaaagtgggtcctaa	Meyer *et al*. 2018
IoT	taattggtaacgaatcagacaattgacggctcgagggagtagcatagggtttgcagaa<br>tccctgcttcgtccatttgacaggcacattatgcatcgatgataagctgtcaaacatg<br>agcagatcctctacgccggacgcatcgtggccggcatcaccggcgccacaggtgcggt<br>tgctggcgcctatatcgccgacatcaccgatggggaagatcgggctcgccacttcggg<br>ctcatgagcaaatattttatctg	Meyer *et al*. 2018
LS32P21	ctcggtaccaaattccagaaaagaggcctcccgaaaggggggccttttttcgttttgg<br>tcc	Meyer *et al*. 2018
rrnbT1	caaataaaacgaaaggctcagtcgaaagactgggcctttcgttttatctgttgtttgt<br>cggtgaacgctctc	Segall-Shapiro *et al.* 2018
ECK120033736	aacgcatgagaaagcccccggaagatcaccttccgggggctttttttattgcgc	Meyer *et al*. 2018

Supplementary Table 4: Plasmids

Plasmid	Description	Source and comments
pxl439	CmR-P$_{UPsp2}$-riboJ-BBa_0032-sfGFP$_d$-rrnbT1-pSC101	
pxl458	CmR-P$_{UPsp2}$-riboJ-BBa_0032-sfGFP$_d$-rrnbT1-pSC101_variant1	
pxl457	CmR-P$_{UPsp2}$-riboJ-BBa_0032-sfGFP$_d$-rrnbT1-pSC101_variant2	
pxl445	CmR-P$_{UPsp2}$-riboJ-BBa_0032-sfGFP$_d$-rrnbT1-pSC101_variant3	
pxl456	CmR-P$_{UPsp2}$-riboJ-BBa_0032-sfGFP$_d$-rrnbT1-pSC101_variant4	

pxl441	CmR-P_{T7A1w3}-SarJ-RBS-TALEsp2-ECK120029600-P_{UPsp2}-riboJ-BBa_0032-sfGFP$_{d}$-rrnbT1-pSC101	
pxl501	CmR-P_{T7A1w3}-SarJ-RBS-TALEsp2-ECK120029600-P_{UPsp2}-riboJ-BBa_0032-sfGFP$_{d}$-rrnbT1-pSC101_variant1	
pxl451	CmR-P_{T7A1w3}-SarJ-RBS-TALEsp2-ECK120029600-P_{UPsp2}-riboJ-BBa_0032-sfGFP$_{d}$-rrnbT1-pSC101_variant3	
pxl452	CmR-P_{T7A1w3}-SarJ-RBS-TALEsp2-ECK120029600-P_{UPsp2}-riboJ-BBa_0032-sfGFP$_{d}$-rrnbT1-pSC101_variant5	
pxl453	CmR-P_{T7A1w3}-SarJ-RBS-TALEsp2-ECK120029600-P_{UPsp2}-riboJ-BBa_0032-sfGFP$_{d}$-rrnbT1-pSC101_variant6	
pxl400	CmR-P_{T7A1w3}-SarJ-RBS-TALEsp2-ECK120029600-P_{UPsp2}-riboJ-BBa_0032-ampR$_{d}$-rrnbT1-pSC101	
pxl497	CmR-P_{T7A1w3}-SarJ-RBS-TALEsp2-ECK120029600-P_{UPsp2}-riboJ-BBa_0032-ampR$_{d}$-rrnbT1-pSC101_variant1	
pxl466	CmR-P_{T7A1w3}-SarJ-RBS-TALEsp2-ECK120029600-P_{UPsp2}-riboJ-BBa_0032-ampR$_{d}$-rrnbT1-pSC101_variant3	
pxl401	CmR-P_{T7A1w3}-SarJ-RBS-TALEsp2-ECK120029600-P_{UPsp2}-riboJ-BBa_0032-ampR$_{d}$-rrnbT1-pSC101_variant5	
pxl419	CmR-P_{T7A1w3}-SarJ-RBS-TALEsp2-ECK120029600-P_{UPsp2}-riboJ-BBa_0032-ampR$_{d}$-rrnbT1-pSC101_variant6	
pxl398	CmR-P_{T7A1w3}-SarJ-RBS-TALEsp2-ECK120029600-P_{UPsp2}-riboJ-BBa_0032-lacI-rrnbT1-pSC101	
pxl480	CmR-P_{T7A1w3}-SarJ-RBS-TALEsp2-ECK120029600-P_{UPsp2}-riboJ-BBa_0032-lacI-rrnbT1-pSC101_variant1	
pxl481	CmR-P_{T7A1w3}-SarJ-RBS-TALEsp2-ECK120029600-P_{UPsp2}-riboJ-BBa_0032-lacI-rrnbT1-pSC101_variant2	
pxl467	CmR-P_{T7A1w3}-SarJ-RBS-TALEsp2-ECK120029600-P_{UPsp2}-riboJ-BBa_0032-lacI-rrnbT1-pSC101_variant3	
pxl431	CmR-P_{T7A1w3}-SarJ-RBS-TALEsp2-ECK120029600-P_{UPsp2}-riboJ-BBa_0032-lacI-rrnbT1-pSC101_variant5	
pTHSSe_60	CmR-P_{T7A1w3}-SarJ-RBS-TALEsp2-ECK120029600-P_{UPsp2}-riboJ-RBSBBa_0032-sfGFP$_{2}$-rrnbT1-pSC101	Segall-Shapiro *et al*. 2018
pxl534	CmR-P_{T7A1w3}-SarJ-RBS-TALEsp2-ECK120029600-P_{UPsp2}-riboJ-RBSBBa_0032-sfGFP$_{2}$-rrnbT1-pSC101_variant1	
pxl470	CmR-P_{T7A1w3}-SarJ-RBS-TALEsp2-ECK120029600-P_{UPsp2}-riboJ-RBSBBa_0032-sfGFP$_{2}$-rrnbT1-pSC101_variant3	
pxl471	CmR-P_{T7A1w3}-SarJ-RBS-TALEsp2-ECK120029600-P_{UPsp2}-riboJ-RBSBBa_0032-sfGFP$_{2}$-rrnbT1-pSC101_variant5	
pxl472	CmR-P_{T7A1w3}-SarJ-RBS-TALEsp2-ECK120029600-P_{UPsp2}-riboJ-RBSBBa_0032-sfGFP$_{2}$-rrnbT1-pSC101_variant6	
pxl444	KanR-P_{BAD}-sfGFP$_{d}$sgRNA-rrnbT1-P_{BAD}-riboJ-BBa_0032-EvolvR-L3S2P21-p15A	
pxl484	KanR-P_{BAD}-ampR$_{d}$sgRNA-rrnbT1-P_{BAD}-riboJ-BBa_0032-EvolvR-L3S2P21-p15A	
pxl417	KanR-$P_{Tac-lacO}$-riboJ-sfGFP$_{1}$-L3S2P21-P_{BAD}-lacIsgRNA-rrnbT1-Pbad-riboJ-BBa_0032-EvolvR-L3S2P21-p15A	
pxl379	KanR-P_{BAD}-lacIsgRNA-rrnbT1-P_{BAD}-riboJ-BBa_0032-EvolvR-L3S2P21-p15A	
pxl597	KanR-P_{BAD}-lacOsgRNA-rrnbT1-P_{BAD}-riboJ-BBa_0032-EvolvR-L3S2P21-P_{BAD}-sarJ-tetR-IoT-p15A	
pxl598	KanR-P_{BAD}-lacIsgRNA-rrnbT1-P_{BAD}-riboJ-BBa_0032-EvolvR-L3S2P21-P_{BAD}-sarJ-tetR-IoT-p15A	
pxl642	CmR-lacO$_{decoy3x}$-$P_{tetO-lacO}$-riboJ-sfGFP$_{2}$-lacI-rrnbT1-pSC101	
pxl643	CmR-lacO$_{decoy3x}$-$P_{tetO-lacO}$-riboJ-sfGFP$_{2}$-lacI-rrnbT1-pSC101_variant1	
pxl644	CmR-lacO$_{decoy3x}$-$P_{tetO-lacO}$-riboJ-sfGFP$_{2}$-lacI-rrnbT1-pSC101_variant2	
pxl645	CmR-lacO$_{decoy3x}$-$P_{tetO-lacO}$-riboJ-sfGFP$_{2}$-lacI-rrnbT1-pSC101_variant3	
pxl646	CmR-lacO$_{decoy3x}$-$P_{tetO-lacO}$-riboJ-sfGFP$_{2}$-lacI-rrnbT1-pSC101_variant4	
pxl639	CmR-lacO$_{decoy3x}$-$P_{tetO-lacO}$-riboJ-sfGFP$_{2}$-lacI-rrnbT1-pSC101_variant5	
pxl665	CmR-lacO$_{decoy3x}$-$P_{tetO-lacO}$-riboJ-sfGFP$_{2}$-lacI-rrnbT1-pSC101_variant6	
pxl652	CmR-lacO$_{decoy3x}$-$P_{tetO-lacO}$-riboJ-sfGFP$_{2}$-ECK120033736-P_{tetO}-lacI-rrnbT1-pSC101	
pxl653	CmR-lacO$_{decoy3x}$-$P_{tetO-lacO}$-riboJ-sfGFP$_{2}$-ECK120033736-P_{tetO}-lacI-rrnbT1-pSC101_variant1	
pxl654	CmR-lacO$_{decoy3x}$-$P_{tetO-lacO}$-riboJ-sfGFP$_{2}$-ECK120033736-P_{tetO}-lacI-rrnbT1-pSC101_variant2	
pxl655	CmR-lacO$_{decoy3x}$-$P_{tetO-lacO}$-riboJ-sfGFP$_{2}$-ECK120033736-P_{tetO}-lacI-rrnbT1-pSC101_variant3	

Note: Unless otherwise stated, plasmids are from this work.

Supplementary Table 5: Key primers

Primer	Sequence	Purpose
P519-BsaI-CCGG-sfGFP-f	TGGTCTCACCGGAAGGCTATGTGCAGGA	Forward primer to create $sfGFP_d$
P520-BsaI-CCGG_RC-sfGFPmut-r	TGGTCTCACCGGTTAGGCGGACTTGAAGAAGTCAT	Reverse primer to create $sfGFP_d$
P479-BsaI-TGAT-amprmut-f	CGGTCTCCTGATAAGATAACACTGCGGCCAACTT	Forward primer to create $ampR_d$
P492-BsaI-TGAT_RC-amprmut-r	CGGTCTCCATCAGGTTATGGCAGCACTGCATA	Reverse primer to create $ampR_d$
P609-BsaI-GCGC-lacO-sgRNAv15-f	CTGGTCTCAGCGCTCACAATTGGTGGAGCGCTCGGT	Building $P_{tetO\text{-}lacO}$
P604-BsaI-GCGC_RC-tetO-r	CTGGTCTCTGCGCTCACAATTCTGCTCAGTATCTCTATCACT	Building $P_{tetO\text{-}lacO}$
P322-BasI-PT3-r	GCGGTCTCCAGCGTTGTTAT	Building $lacO_{decoy}$-$P_{tetO\text{-}lacO}$
P111-Kan-f	GACGTTTCCCGTTGAATATGGCT	Building $lacO_{decoy}$-$P_{tetO\text{-}lacO}$
P610-BsaI-GCAC-tet1(lac1)-f	CTGGTCTCTGCACGGAAGAGAGTCAATTCAGGGT	Building P_{tetO}-lacI module
P616-BsaI-GCAC_RC-ptetO1-r3	CTGGTCTCCGTGCATTATATCTCTATCACTGATAGGGATGTCAACGA	Building P_{tetO}-lacI module

Note: BsaI recognition site, 4 bp sticky end.

Supplementary Table 6: sgRNA characterization

sgRNA	CRISPRscan score	free energy (kcal/mol)
sgRNA targeting $sfGFP_d$	44	-27.63
sgRNA targeting $ampR_d$	38	-32.83
sgRNA targeting *lacI*	44	-35.99
sgRNA targeting *lacO*	68	-38.50

Note: CRISPRscan scores are obtained by submitting the sequences of $sfGFP_d$, $ampR_d$, *lacI*, and $P_{tetO\text{-}lacO}$ from the pSC101 plasmids to the CRISPRscan web server, without specifying either the genome or the promoter. The efficiency scores can be found for the corresponding targeting regions on the pSC101 plasmids. The free energy is calculated with the ViennaRNA web server, using the sgRNA seed sequence together with the scaffold from the corresponding p15A plasmids (i.e., pxl444, pxl484, pxl598 and pxl597).

References

[1] Halperin, S. O. *et al.* CRISPR-guided DNA polymerases enable diversification of all nucleotides in a tunable window. *Nature* **560**, 248–252 (2018).

[2] Krašovec, R. *et al.* Measuring microbial mutation rates with the fluctuation assay. *Journal of Visualized Experiments* 60406 (2019).

[3] Guzman, L. M., Belin, D., Carson, M. J. & Beckwith, J. Tight regulation, modulation, and high-level expression by vectors containing the arabinose pbad promoter. *Journal of Bacteriology* **177**, 4121–4130 (1995).

[4] Klein, M., Eslami-Mossallam, B., Arroyo, D. G. & Depken, M. Hybridization kinetics explains crispr-cas off-targeting rules. *Cell Reports* **22**, 1413–1423 (2018).

[5] Jones, D. L. *et al.* Kinetics of dcas9 target search in escherichia coli. *Science* **357**, 1420–1424 (2017).

[6] Drake, J. W. A constant rate of spontaneous mutation in dna-based microbes. *Proceedings of the National Academy of Sciences* **88**, 7160–7164 (1991).

[7] Shao, B. *et al.* Single-cell measurement of plasmid copy number and promoter activity. *Nature Communications* **12**, 1475 (2021).

[8] Verrou, K.-M., Pavlopoulos, G. A. & Moulos, P. Protocol for unbiased, consolidated variant calling from whole exome sequencing data. *STAR Protocols* **3**, 101418 (2022).

[9] Castillo-Hair, S. M. *et al.* FlowCal: A User-Friendly, Open Source Software Tool for Automatically Converting Flow Cytometry Data from Arbitrary to Calibrated Units. *ACS Synthetic Biology* **5**, 774–780 (2016).

[10] Erez, A., Vogel, R., Mugler, A., Belmonte, A. & Altan-Bonnet, G. Modeling of cytometry data in logarithmic space: When is a bimodal distribution not bimodal? *Cytometry Part A* **93**, 611–619 (2018).

[11] Beal, J. *Flow Cytometry Quantification of Transient Transfections in Mammalian Cells*, 153–176 (Springer US, New York, NY, 2024).

[12] Mazoyer, A., Drouilhet, R., Despréaux, S. & Ycart, B. flan: An R Package for Inference on Mutation Models. *The R Journal* **9**, 334–351 (2017).

[13] Segall-Shapiro, T. H., Sontag, E. D. & Voigt, C. A. Engineered promoters enable constant gene expression at any copy number in bacteria. *Nature Biotechnology* **36**, 352–358 (2018).

[14] Gyorgy, A., Menezes, A. & Arcak, M. A blueprint for a synthetic genetic feedback optimizer. *Nature Communications* **14**, 2554 (2023).

[15] Salis, H. M. Chapter two - the ribosome binding site calculator. In Voigt, C. (ed.) *Synthetic Biology, Part B*, vol. 498 of *Methods in Enzymology*, 19–42 (Academic Press, 2011).

[16] Igler, C., Lagator, M., Tkacik, G., Bollback, J. P. & Guet, C. C. Evolutionary potential of transcription factors for gene regulatory rewiring. *Nature Ecology & Evolution* **2**, 1633–1643 (2018).

[17] Gyorgy, A. Competition and evolutionary selection among core regulatory motifs in gene expression control. *Nature Communications* **14**, 8266 (2023).

[18] Meyer, A. J., Segall-Shapiro, T. H., Glassey, E., Zhang, J. & Voigt, C. A. Escherichia coli "marionette" strains with 12 highly optimized small-molecule sensors. *Nature Chemical Biology* **15**, 196–204 (2019).

[19] Lee, J. W. *et al.* Creating single-copy genetic circuits. *Molecular Cell* **63**, 329–336 (2016).

[20] Huang, D., Holtz, W. J. & Maharbiz, M. M. A genetic bistable switch utilizing nonlinear protein degradation. *Journal of Biological Engineering* **6**, 9 (2012).

[21] Daeffler, K. N. *et al.* Engineering bacterial thiosulfate and tetrathionate sensors for detecting gut inflammation. *Molecular Systems Biology* **13**, 923 (2017).
