# Supplementary material for: Tuning evolvability via plasmid copy number and regulatory architecture
Source: Nat Commun. 2025 Dec 31;17:1230. doi: 10.1038/s41467-025-67995-9 (PMC12864740; doi:10.1038/s41467-025-67995-9)
Supplement: Supplementary file 2 — Reporting Summary [file 41467_2025_67995_MOESM2_ESM.pdf]

nature portfolio

Corresponding author(s): Andras Gyorgy

Last updated by author(s): December 11, 2025

Reporting Summary

Nature Portfolio wishes to improve the reproducibility of the work that we publish. This form provides structure for consistency and transparency in reporting. For further information on Nature Portfolio policies, see our Editorial Policies and the Editorial Policy Checklist.

Statistics

For all statistical analyses, confirm that the following items are present in the figure legend, table legend, main text, or Methods section.

n/a	Confirmed	
☐	☒	The exact sample size (*n*) for each experimental group/condition, given as a discrete number and unit of measurement
☐	☒	A statement on whether measurements were taken from distinct samples or whether the same sample was measured repeatedly
☐	☒	The statistical test(s) used AND whether they are one- or two-sided<br>*Only common tests should be described solely by name; describe more complex techniques in the Methods section.*
☒	☐	A description of all covariates tested
☐	☒	A description of any assumptions or corrections, such as tests of normality and adjustment for multiple comparisons
☐	☒	A full description of the statistical parameters including central tendency (e.g. means) or other basic estimates (e.g. regression coefficient) AND variation (e.g. standard deviation) or associated estimates of uncertainty (e.g. confidence intervals)
☐	☒	For null hypothesis testing, the test statistic (e.g. F, t, r) with confidence intervals, effect sizes, degrees of freedom and P value noted<br>*Give P values as exact values whenever suitable.*
☒	☐	For Bayesian analysis, information on the choice of priors and Markov chain Monte Carlo settings
☒	☐	For hierarchical and complex designs, identification of the appropriate level for tests and full reporting of outcomes
☒	☐	Estimates of effect sizes (e.g. Cohen's d, Pearson's r), indicating how they were calculated

Our web collection on statistics for biologists contains articles on many of the points above.

Software and code

Policy information about availability of computer code

Data collection	The AttuneTM NxT Software (version 5.2.0) was used for acquisition of flow cytometry data from the Attune NxT Flow Cytometer. BD FACSDiva Software (version 9.4) was used for cell sorting from BD Aria III Sorter.
Data analysis	For data analysis and visualization, Python (version 3.10.14) was used in the JupyterLab interface (version 4.2.2), source code is available at https://doi.org/10.5281/zenodo.17642376. FlowCal python library (version 1.3.0) was used to analyze flow cytometry data. R (version 4.1.2) and flan package (version 0.9) were used for fluctuation analysis. Adobe Illustrator (version 28.5) was used for creating figures. Microsoft Word for Mac (version 16.62) was used to create the tables presented in the Supplementary Information. Overleaf (version v2) online LaTeX editor was used to prepare and compile the manuscript.

For manuscripts utilizing custom algorithms or software that are central to the research but not yet described in published literature, software must be made available to editors and reviewers. We strongly encourage code deposition in a community repository (e.g. GitHub). See the Nature Portfolio guidelines for submitting code & software for further information.

Data

Policy information about availability of data

All manuscripts must include a data availability statement. This statement should provide the following information, where applicable:

- Accession codes, unique identifiers, or web links for publicly available datasets
- A description of any restrictions on data availability
- For clinical datasets or third party data, please ensure that the statement adheres to our policy

Source Data are provided with this paper. Sequencing data generated in this study are uploaded to NCBI SRA (PRJNA1373182).

Research involving human participants, their data, or biological material

Policy information about studies with human participants or human data. See also policy information about sex, gender (identity/presentation), and sexual orientation and race, ethnicity and racism.

Reporting on sex and gender	N/A
Reporting on race, ethnicity, or other socially relevant groupings	N/A
Population characteristics	N/A
Recruitment	N/A
Ethics oversight	N/A

Note that full information on the approval of the study protocol must also be provided in the manuscript.

Field-specific reporting

Please select the one below that is the best fit for your research. If you are not sure, read the appropriate sections before making your selection.

☒ Life sciences ☐ Behavioural & social sciences ☐ Ecological, evolutionary & environmental sciences

For a reference copy of the document with all sections, see nature.com/documents/nr-reporting-summary-flat.pdf

Life sciences study design

All studies must disclose on these points even when the disclosure is negative.

Sample size	For all experiments, a minimum of three biological replicates were analyzed for each condition. The sample size was determined to be sufficient to detect statistical differences between conditions.
Data exclusions	No data were excluded from the analysis.
Replication	Experiments were replicated at least twice and with similar results obtained.
Randomization	Groups were defined entirely by predetermined genetic constructs and controlled experimental conditions rather than by assigning biological samples to treatment arms. Covariates such as growth medium, temperature, culture handling, and induction protocols were strictly standardized across all groups, and data were collected using automated, unbiased measurement platforms. Because group identity is intrinsic and engineered, and external variables were tightly controlled, random allocation of samples was not applicable in this study.
Blinding	All experiments involved objectively measurable phenotypes generated by bacteria harboring engineered genetic constructs. Group identities (e.g., PCN variants and synthetic gene circuits) are intrinsic, unambiguous features encoded in the constructs themselves and cannot be altered or obscured. Data collection and computational analysis were performed using automated instrumentation and pipelines with quantitative metrics, eliminating observer bias. Therefore, blinding was not applicable to this work.

Reporting for specific materials, systems and methods

We require information from authors about some types of materials, experimental systems and methods used in many studies. Here, indicate whether each material, system or method listed is relevant to your study. If you are not sure if a list item applies to your research, read the appropriate section before selecting a response.

Materials & experimental systems

n/a	Involved in the study
☒	☐ Antibodies
☒	☐ Eukaryotic cell lines
☒	☐ Palaeontology and archaeology
☒	☐ Animals and other organisms
☒	☐ Clinical data
☒	☐ Dual use research of concern
☒	☐ Plants

Methods

n/a	Involved in the study
☒	☐ ChIP-seq
☐	☒ Flow cytometry
☒	☐ MRI-based neuroimaging

Plants

Seed stocks: N/A

Novel plant genotypes: N/A

Authentication: N/A

Flow Cytometry

Plots

Confirm that:

☒ The axis labels state the marker and fluorochrome used (e.g. CD4-FITC).

☒ The axis scales are clearly visible. Include numbers along axes only for bottom left plot of group (a 'group' is an analysis of identical markers).

☒ All plots are contour plots with outliers or pseudocolor plots.

☒ A numerical value for number of cells or percentage (with statistics) is provided.

Methodology

Sample preparation: Samples were prepared and processed in LB culture in exponential phase.

Instrument: Attune NxT Flow Cytometer and BD FACSAria III Sorter.

Software: Attune™ NxT Software (version 5.2.0) and BD FACSDiva Software (version 9.4).

Cell population abundance: 100,000 total events per sample for analysis using Attune and at least 1000 events collected for sorting using BD Aria III.

Gating strategy: Cells were gated according to forward scatter height (FSC-H) and side scatter height (SSC-H).

☒ Tick this box to confirm that a figure exemplifying the gating strategy is provided in the Supplementary Information.
